# Post-myocardial infarction fibrosis: Pathophysiology, examination, and intervention

**DOI:** 10.3389/fphar.2023.1070973

**Published:** 2023-03-28

**Authors:** Xiaoying Yin, Xinxin Yin, Xin Pan, Jingyu Zhang, Xinhui Fan, Jiaxin Li, Xiaoxuan Zhai, Lijun Jiang, Panpan Hao, Jiali Wang, Yuguo Chen

**Affiliations:** ^1^ Department of Emergency and Chest Pain Center, Qilu Hospital of Shandong University, Jinan, China; ^2^ Clinical Research Center for Emergency and Critical Care Medicine of Shandong Province, Institute of Emergency and Critical Care Medicine of Shandong University, Qilu Hospital of Shandong University, Jinan, China; ^3^ Key Laboratory of Emergency and Critical Care Medicine of Shandong Province, Qilu Hospital of Shandong University, Jinan, China; ^4^ Key Laboratory of Cardiovascular Remodeling and Function Research, Chinese Ministry of Education, Chinese Ministry of Health and Chinese Academy of Medical Sciences, The State and Shandong Province Joint Key Laboratory of Translational Cardiovascular Medicine, Qilu Hospital of Shandong University, Jinan, China

**Keywords:** myocardial infarction, fibrosis, cardiac remodeling, antifibrotic therapy, extracellular matrix, fibroblast, myofibroblast

## Abstract

Cardiac fibrosis plays an indispensable role in cardiac tissue homeostasis and repair after myocardial infarction (MI). The cardiac fibroblast-to-myofibroblast differentiation and extracellular matrix collagen deposition are the hallmarks of cardiac fibrosis, which are modulated by multiple signaling pathways and various types of cells in time-dependent manners. Our understanding of the development of cardiac fibrosis after MI has evolved in basic and clinical researches, and the regulation of fibrotic remodeling may facilitate novel diagnostic and therapeutic strategies, and finally improve outcomes. Here, we aim to elaborate pathophysiology, examination and intervention of cardiac fibrosis after MI.

## 1 Introduction

Myocardial infarction (MI) is a leading cause of global morbidity and mortality and the primary contributor to heart failure (HF) (Groenewegen et al., 2020). The limited regenerative capacity leads to massive loss of cardiomyocytes (CMs) and excessive deposition of extracellular matrix (ECM) after MI (Prabhu and Frangogiannis, 2016; Dattagupta and Immaneni, 2018), which is called cardiac remodeling. Cardiac fibrosis is a pathological process of cardiac remodeling. Although timely and effective reperfusion can reverse this adverse effect, the incidence of cardiac fibrosis is increasing. During a life span of post-MI patients, fibrotic tissue accumulates in the process of left ventricular remodeling, and expands over time to remote non-infarcted region, which significantly alters cardiac structure and deteriorates cardiac function (Gil et al., 2022). Many patients survive with long-term adverse prognosis created strain on already overstretched healthcare systems and hampered medical management.

Abundant interstitial and perivascular fibroblasts in the adult heart play an essential role in maladaptive repair and fibrosis. Resident cardiac fibroblasts (CFs) are considered the primary cells that maintain ECM homeostasis by overseeing its quantity and quality, even if they are activated by pathological signals. To prevent the catastrophic outcomes of MI, CFs and myofibroblasts (MFs) deposit ECM to replace necrotic CMs and maintain the structural integrity of the heart coming along with viable CMs hypertrophy, whereas excessive ECM accumulation forms a fibrotic scar that provokes cardiac dysfunction and lethal arrhythmias (Burke et al., 2021a). Moreover, cardiac systolic dysfunction can be induced *via* scar with low tensile strength after disordered healing, while diastolic dysfunction increases after excessively fibrogenic activation and collagen deposition (Venugopal et al., 2022). Unfortunately, interventions for post-MI fibrotic remodeling have been limited.

With the limitation of therapeutic effects of drugs and surgeries, cardiac fibrosis is normally in MI patients. Despite various studies now addressing myocardial fibrosis, the understanding of its pathogenesis, clinical implications, and managements remains limited.

## 2 Pathophysiology of post-MI cardiac fibrosis

Fibrosis is a crucial determinant of cardiac function, stiffness, and conduction, with cardiac elasticity and compliance decreasing as fibrosis increases, contributing to systolic and diastolic dysfunction and even lethal arrhythmia and impairment of oxygen utilization (Fan et al., 2012). Thereby, it is very important to note that the complex pathophysiology and multiple mechanisms have been implicated in the fibrotic process following AMI. Also, the related molecular signaling network is complex and sophisticated, which involves various inflammatory mediators, inflammatory cells, and activating stromal fibrogenic effector cells, such as fibroblasts (Rockey et al., 2015). Additionally, the cardiac stromal cells exert profibrotic action *via* secreting cardiokines, which can predict adverse fibrotic remodeling after MI. (Masurkar et al., 2023).

The post-MI remodeling has three phases: the inflammatory phase (the first 3 days), proliferative phase (3–14days), and maturation phase (2weeks–2months) accompanying the dilation of non-infarcted zone, hypertrophy of CMs, and phenotypic transformation of CFs (Venugopal et al., 2022). During cardiac remodeling, inflammation, oxidative stress, disordered ECM, and CFs collectively cause cardiac fibrosis. According to the features and location of ECM protein deposition, there are usually two species of post-MI fibrosis: reparative and reactive fibrosis, with the former directly replacing necrotic cardiac tissue after MI and the latter being the pathological consequence of over-activated CFs and including perivascular and interstitial fibrosis (de Boer et al., 2019). Since adverse events and mortality are regarded as being related to the severity of cardiac fibrosis (Benjamin et al., 2018), uncovering these mechanisms is critical to help source novel therapeutic targets, diagnostic or prognostic performance.

### 2.1 Fibroblasts and myofibroblasts

Noteworthily, there are relatively static CFs and no MFs in a healthy heart (Hall et al., 2021). Recent single-cell multi-omics studies have elevated our knowledge of CFs in cardiac fibrosis (Forte et al., 2021a). For example, new CFs have been found, presenting as early as 1 day after MI (Shi et al., 2021), which can promote inflammation and recruit leukocytes. Then, CFs transfer to a proliferative, reparative, and proangiogenic phenotype, with maximum proliferation within 2–4 days. Moreover, the DNA damage response-associated CFs are up-regulated from day 3 up to day 7, as well as some senescence-associated CFs at day 7, indicating that cardiac fibrosis can be limited *via* activating DNA damage response and senescence (Shibamoto et al., 2019). Finally, CFs downregulate angiogenesis and convert to MFs at week 1 (Mouton et al., 2019) under new baseline conditions that the ultimate profibrotic culprits MFs produce excessive ECM, consisting principally of collagen I and III accompanying proteoglycans and elastin fibers (Kruszewska et al., 2022). Interestingly, although collagen V is minimally expressed, its deficiency increases scar size and cardiac dysfunction in an MI mouse model (Yokota et al., 2020). Recent research also finds that CFs interact with platelet leading to the alteration of collagen composition and content, and platelets mediate the reduction of inflammation after 24 h and scar formation after 21 days post AMI (Reusswig et al., 2022).

In a fibroblast-ablated mice model, there is a pronounced downregulation of CFs and the network formed by the collagen VI, microfibrillar collagen and the basement membrane, without accompanying overtly alteration of fibrillar collagen and the ECM proteome. Surprisingly, cardiac function is better preserved after MI, which suggests that controlled fibroblast reduction may have cardioprotective and therapeutic value in heart disease (Kuwabara et al., 2022). Moreover, as the mainly producers of ECM, CFs also produce cytokines, together with macrophages (Blythe et al., 2019), while, inactivating transcription factor sex-determining region Y box nine in CFs reduces cardiac fibrosis and late inflammation (Scharf et al., 2019). Thus, it is very important to distinguish the various phenotypes and functions of CFs under different conditions through detecting the markers of CFs. For instance, a study has identified proangiogenic and fibroblast-specific protein 1 (FSP1)-positive CFs that were distinct from profibrotic MFs (Saraswati et al., 2019). Moreover, CFs with growth factor receptor platelet-derived growth factor receptor α (PDGFRα) deficiency are responsible for the 50% reduction in CFs quantity (Ivey et al., 2019), while CFs with smad3 deficiency produce a decreased level of collagen (Huang et al., 2020). Further, there are several other markers of CFs, such as vimentin, transcription factor Tcf21, and MEFSK4 (mouse embryonic fibroblasts) (Venugopal et al., 2022). However, except for Tcf21 and PDGFRα, these markers are insufficiently sensitive and due to a lack of specific features (Acharya et al., 2012; Alex et al., 2022).

MFs have the specific markers as well, such as periostin and α-smooth muscle actin (α-SMA) (Kanisicak et al., 2016; Venugopal et al., 2022). In addition, after 1 week of MI, MFs increase in number and express periostin, collagen triple helix repeat containing 1, and dimethylarginine dimethylaminohydrolase 1, while the last is also expressed in activated CFs (Zhuang et al., 2020). Further, MFs are heterogeneous, i.e., they have different phenotypes; for instance, some are proliferating cells, and others express different levels of transforming growth factor β1 (TGF-β1), thrombospondin 4, and periostin (Farbehi et al., 2019).

There is, therefore, a clear and pressing need to identify additional novel mediators of cardiac fibrosis presentation and progression in response to pathological stimuli to facilitate the development of alternative therapeutic strategies targeting cardiac fibrosis.

During the process of cardiac fibrosis, CFs and CF-to-MF transformation are pathologically activated by many damage stimuli, such as TGF-β, platelet-derived growth factor (PDGF), epidermal growth factor (EGF), fibroblast growth factor (FGF), tumor necrosis factor α (TNF-α), angiotensin II (Ang II), interleukin-1 (IL-1), IL-4, and aldosterone (Fan et al., 2012). Recent research finds G-protein-coupled receptor kinase (GRK)-5 regulates fibroblast activation *in vitro* and *in vivo*, which suggests the inhibitor of GRK5 may be a novel target (Eguchi et al., 2021).

### 2.2 Extracellular matrix

In the working hearts, the relative glide movement of CMs and blood flow generates shear forces, constituting mechanical force with stretch and strain constrains, which is an important regulator of cells activation in fibrosis. ECM provides the heart with a structural scaffold and interacts with cells *via* adhesion molecules, such as integrins and cadherins, and distributes mechanical force through the cardiac tissue to individual cell (Souders et al., 2009; Zheng et al., 2016). In brief, collagen binding is responsible for load transfer and unnormal stretching limitation (Weber et al., 1994), which suggests the amount, distribution, and organization of ECM components modulate cardiac morphology and function (Spinale, 2007; Chute et al., 2019). For example, a pure scar in a rat MI model has lower wall thickness reduction over time before and after the decellularization, indicating excess collagen deposition during scar maturation and overall stiffening (Brazile et al., 2021). Moreover, the CMs fusion significantly decreases along with massive CMs death producing space and unnormal mechanical forces, which alters activation patterns of the cells and promotes CF-to-MF transformation (Kachanova et al., 2022).

The production and degradation of ECM are commonly regulated by MFs, together with macrophages and other cell types (Yap et al., 2023), such as CFs produce matrix metalloproteinases (MMPs) degrading collagens and tissue inhibitors of MMPs (TIMPs) inducing collagen synthesis (Nikolov and Popovski, 2022). There are four major ECM-associated peptides: the N-terminal propeptide of collagen type III (PIIINP) (indicating collagen III synthesis), the C-terminal telopeptide of collagen type I (ICTP) (indicating collagen I degradation), N-terminal propeptide of collagen type I (PINP), and C-terminal propeptide of collagen type I (PICP) (both PINP and PICP indicate collagen I synthesis) (Nikolov and Popovski, 2022). A recent study finds that the single mutation of thioredoxin-interacting protein cysteine 247 reduces collagen I α1 chain in MI mice (Nakayama et al., 2021). During scar formation, optimal collagen crosslinks require disintegrin and metalloproteinases (Chute et al., 2022), whereas PXS-5153A, the lysyl oxidase-like 2/3 enzymatic inhibitor, reduces collagen crosslinking and fibrosis (Schilter et al., 2019). Additionally, anti-integrin α(v) therapy also diminishes cardiac fibrosis *via* suppressing integrin-ECM interactions and cell adhesin (Bouvet et al., 2020). Also, there are several other fibrosis-associated non-collagen components, such as osteopontin, periostin, and galectin-3 (Li et al., 2022b).

The imbalance of ECM production and degradation induces adverse cardiac remodeling and dysfunction, with the insufficient repair disrupting cardiac tissue integrity, and excessive scar diminishing therapeutic efficacy (Yap et al., 2023).

### 2.3 Neuroendocrine system

#### 2.3.1 Renin-angiotensin-aldosterone system

The activation of renin-angiotensin-aldosterone system (RAAS) plays a crucial role in development and progression of MI (Shigemura et al., 2019), having independently association with a higher risk of adverse cardiovascular events and mortality (Ivanes et al., 2012). After MI, the cardiac overload causes chamber dilation and elevates cardiac wall stress, followed by activating RAAS and inflammation response, which promotes the formation of MFs and excessive fibrosis (Fan and Guan, 2016; Zhou et al., 2019).

The essential process of RAAS is activated as described below. As the initial and rate-limiting step of the classical RAAS, renin is an aspartyl protease mainly produced by the juxtaglomerular cells of the renal afferent arteriole (Shigemura et al., 2019). Its plasma activity is associated with greater burden of coronary artery disease (Unkart et al., 2020). The synthesis and release of renin are stimulated by three major mechanisms: the decrease of sodium chloride concentration in the macula dense and perfusion pressure as sensed by renal baroreceptors; and the activation of β-adrenergic receptors in juxtaglomerular cells by catecholamines (Gomez and Sequeira-Lopez, 2018).

The liver produces angiotensinogen in the circulation, which is then activated by renin in juxtaglomerular cells to generate inactive angiotensin I (Ang I) (AlQudah et al., 2020). Subsequently, angiotensin-converting enzyme (ACE) converts Ang I into biologically active octapeptide Ang II (AlQudah et al., 2020), which is degraded to angiotensin III and several other angiotensins (Boorsma et al., 2021). For example, Ang II can be converted to angiotensin (1–7) *via* ACE2 with subsequently activating Mas receptor to decrease myocardial fibrosis (Boorsma et al., 2021), alternatively converted to Ang III with binding type 1 receptor by aminopeptidase A (Boitard et al., 2019). Moreover, both Ang II and Ang (1–7) are cleaved enzymatically to Ang (3–7) and Ang (5–7) *vi*a fibroblast growth factor-23 (FGF-23) stimulating dipeptidyl peptidase 3, with countering the therapeutic benefits from angiotensin-converting enzyme inhibitors (ACEI) and angiotensin receptor blockers (ARB) binding Mas receptor, which can be suppressed *via* the specific dipeptidyl peptidase three antibody procizumab (Boorsma et al., 2021). Interestingly, Ang II stimulates osteopontin synthesis and increases the concentrations of FGF-23 to negatively regulate ACE2 concentrations (Boorsma et al., 2021). As the main RAAS effector peptide, Ang II usually has 2 G protein-coupled receptors (GPCRs): type 1 Ang II receptor (AT1R) and the type 2 receptor (AT2R) (Pugliese et al., 2020), with the activation of the former increasing proinflammatory response and aldosterone levels, and the later having anti-fibrotic and anti-inflammatory effects (Ziaja et al., 2021). At the meantime, Ang II causes CMs hypertrophy, CFs hyperplasia (Leancă et al., 2022), and the secretion of molecular mediators (e.g., norepinephrine and endothelin) to promote cardiac remodeling (Williams, 2001; Gajarsa and Kloner, 2011). For example, endothelin 1 (ET-1), a potent vasoconstrictor peptide, promotes inflammatory and CMs hypertrophy to cause adverse remodeling (Zhang et al., 2019a; Haryono et al., 2022). Interestingly, alamandine, a substance with only one amino acid residue difference from Ang II, alleviates cardiac dysfunction and fibrosis *via* inhibiting oxidative stress *in vivo* and vitro models (Zhao et al., 2022a). In addition, the upregulation of left ventricle voltage-dependent anion channel one in MI patients is regulated *via* the RAAS activation, and inhibition of the anion channel reduces atrial fibrosis (Klapper-Goldstein et al., 2020).

The regulation of RAAS is relative with multiple signaling pathways (e.g., extracellular signal-regulated kinase (ERK) and janus kinase (JAK) pathways) (Chen et al., 2020b); for instance, RAAS promotes collagen secretion *via* TGF-β1/smad2/3 pathway (Li et al., 2022b). Moreover, microRNA (miRNA) can also regulate RAAS, such as miR-181/Adamts1/neutrophil gelatinase-associated lipocalin pathway (Garg et al., 2020). Further, Ang II promotes CFs viability, activation, and migration through the circCELF1/miR-636/dickkopf WNT signaling pathway inhibitor 2 pathway in AMI mice (Li et al., 2022g).

#### 2.3.2 Sympathetic nervous system

After AMI, sympathetic nervous system (SNS) is activated immediately as a compensatory mechanism to increase cardiac output and maintain blood pressure. The norepinephrine is primarily synthesized and secreted from adrenal medulla and is modulated through β-adrenergic receptors (β-ARs) on the heart (Lymperopoulos et al., 2007).

The heart expresses various ARs belonging to GPCRs that the most predominant subtype belongs to β1-AR, 15% to β2-AR, and the remainder to β3-AR and α1-AR. Briefly, β-ARs regulate cardiac function *via* impacting myocardial contractility (Tanner et al., 2021), such as β1-AR and β2-AR with chronotropic and ionotropic effects, contrarily β3-AR with negative inotropic properties (Yoshikawa et al., 1996; Capote et al., 2015; Lymperopoulos, 2018). Noteworthily, the β2-AR, not the β1-AR, is the predominant subtype in the non-cardiocytes (e.g., fibroblasts, endothelial cells, immune cells) (Lymperopoulos et al., 2021). For example, CMs death, hypertrophy, and cardiac fibrosis are decreased in β2-AR knockout bone marrow transplantation mice following isoproterenol treatment, which suggests β2-AR expresses in the heart’s immune cells (Atsuki et al., 2019; Tanner et al., 2021). As a GPCR, β2-AR couples to both Gs and Gi proteins, and the β2-AR-stimulated cardioprotective Gi signaling depends on the heterodimerization of β2-ARs and 5-hydroxytryptamine receptors 2B (Song et al., 2021c).

Recent years, β3-AR has become a therapeutic target for cardiac fibrosis. Mechanistically, the reduced reactive oxygen species (ROS) levels following β3-AR activation attenuate fibrosis through reduced release of paracrine profibrotic agents in β3-AR expressing myocytes. Moreover, β3-AR/protein kinase G (PKG) signaling emerged as a promising therapeutic target in heart failure with preserved ejection fraction (HFpEF). Diastolic dysfunction in patients with HFpEF mainly results from the combination of increased cardiomyocyte stiffness with left ventricle (LV) hypertrophic remodeling and interstitial fibrosis. Cardiomyocyte stiffness results from both increased myofilaments Ca^2+^ sensitivity and higher titin stiffness, related to reduced PKG activity in the myocardium of HFpEF patients and subsequent lower phosphorylation of these targets. Therefore, β3-AR stimulation, by improving nitric oxide synthase (NOS)/PKG signaling, should restore the phosphorylation of sarcomeric proteins but also improve the regulation of Ca^2+^ handling for cardiac myocytes relaxation (Michel et al., 2020). For example, treatment with β3-AR agonists can potentially address this because they stimulate cardiac myocyte Na^+^/K^+^-ATPase. This occurs *via* downstream activation of cyclic guanosine monophosphate-dependent signaling pathways which the β3-AR has in common with NO donors, GC-1 activators15 and the angiotensin receptor-neprilysin inhibitor (ARNI) (Bundgaard et al., 2022).

After MI, upregulated SNS activity induces hematopoietic stem and progenitor cells proliferation and release in the bone marrow. In the early stage, sympathetic overdrive activates apoptotic pathway and promotes neutrophil influx in the necrotic area and infarct expansion (Amin et al., 2011). Subsequently, the stimulation of β1-AR promotes CMs hypertrophy and renin release (Gajarsa and Kloner, 2011). In MI mice, exchange protein activated by cyclic-adenosine, which could be upregulated by β-AR activation, prevents left atrial fibrosis (Surinkaew et al., 2019), while β-AR mainly modulates collagen production by CFs and causes proliferation of human CFs and fibrotic remodeling (Turner et al., 2003; Humeres and Frangogiannis, 2019). Further, β2-AR upregulates CFs proliferation *via* the secretion of IL-6, depending on Gαs/ERK1/2 (Tanner et al., 2020). To sum up, the regulation of sympathetic neurohormone expression is a key therapeutic option in attenuating cardiac remodeling.

#### 2.3.3 Natriuretic peptides

Natriuretic peptides (NPs), primarily as endocrine hormones, are secreted by atrial and ventricular CMs under upregulated wall stress and stretching in MI and regulate diuresis, natriuresis, and vasodilation, as well as inhibit SNS, RAAS, and ET-1 (Kuhn, 2016). NPs have three isoforms: A-type NP (ANP, which inhibits collagen synthesis and is a main fibrotic driver), B-type NP (BNP; a prognosis predictor after MI), and C-type NP (CNP) (Kangawa et al., 1984; Kuhn, 2016). NPs have anti-apoptotic, anti-inflammatory, and anti-fibrotic effects to prevent myocardial ischemic reperfusion injury (MIRI) and adverse remodeling, with their concentrations reflecting profibrotic environments and identifying patients at risk for remodeling, while ANP and BNP reduce vasoconstriction, CFs proliferation, CF-to-MF transformation, collagen synthesis, and MMP release by activating the cyclic guanosine monophosphate (cGMP) pathway (Goetze et al., 2020). The inhibition of phosphodiesterase-9 activity dose-dependently increases cGMP and the cGMP/NP ratio of plasma and urinary, which suggests that its inhibition may constitute a novel therapeutic approach in clinical HF. (Scott et al., 2019).

### 2.4 Immunity

The immune system plays an important role in maintaining homeostasis after MI, in which dying CMs release many damage-associated molecular patterns (DAMPs) and activate the cascade of inflammatory mediators (e.g., inflammatory cytokines and chemokines) (Simões and Riley, 2022). Noteworthily, the severity of inflammatory responses dictates the degree of MI (Ong et al., 2018). Thus, it is important to strike an appropriate balance between inflammatory and anti-inflammatory response, and timely regulate inflammatory signals during cardiac remodeling (Yap et al., 2023). After MI, immune response is rapidly initiated *via* mobilizing distinct immune cell populations (Yap et al., 2023). The differentially altering immune cells infiltration affects cardiac fibrosis, for instance, allografts suppression of tumorigenicity 2 (ST-2) deficiency reduces infiltration of F4/80 (+) macrophages, CD3 (+) T cells and CD20 (+) B cells and thus alleviates vascular occlusion and fibrosis of allografts (Zhang et al., 2021e). In this section, we focus on different immune cell populations, cytokines, chemokines and growth factors to improve mechanistic understanding of immune responses. Then, later on in this article, we will focus in particular on preventing fibrosis with immune treatment. In what follows, we first describe the key features of different immune cells.

#### 2.4.1 Neutrophils and macrophages

As the most abundant peripheral blood granulocytes, neutrophils constitute 50%–70% of the total amount of blood granulocytes in most mammals and 20%–30% in mice (Mestas and Hughes, 2004) in a physiologically circadian pattern (Aziz et al., 2021), exiting blood with night-time peaking (fresh neutrophils) and day-time peaking (aged neutrophils) (Adrover et al., 2019). Recent studies have shown that neutrophils modulate cardiac remodeling *via* the initiation and termination of an inflammatory response and largely infiltrate the infarct zone 1 day after MI and the peri-infarct zone 4 weeks after MI, with disturbed circadian rhythm, in mice (Zhong et al., 2022). However, neutrophil-mediated MIRI is limited by selectin-targeting glycosaminoglycan-peptide conjugate (Dehghani et al., 2022). Normally, massive leucocytes remain in the bone marrow, and only a low number of leucocytes stay in the circulation, whereas 2-arachidonoylglycerol induces massive cardiac infiltration by neutrophils and monocytes and increases cardiac dysfunction and fibrosis in a MIRI mouse model (Schloss et al., 2019). In MI patients, immature CD10^neg^ neutrophils and CD14+HLA-DR^neg/low^ monocytes increase in number (Fraccarollo et al., 2021), and there are still around 10-fold more leukocytes in the scar than in remote zone at 6 weeks after MI in mice (Scharf et al., 2019).

Recent evidence has associated monocytes/macrophages with the etiopathology of cardiac fibrosis, and the interventions targeting these cells have been challenging due to the heterogeneity and the antagonizing roles of different subtypes (Chen et al., 2022b). Remarkably, as the largest population of immune cells, macrophages can be classified into two general subtypes based on cell surface with or without C-C chemokine receptor 2 (CCR2): monocyte-derived CCR2+ macrophages, and yolk-sac-derived CCR2– macrophages. The former exerts pro-inflammatory effects and recruits monocytes, whereas the latter is considered to prevent excessive inflammation (Dick et al., 2019; Yap et al., 2023). At the meantime, macrophages also can be classified as classically activated (M1) or alternatively activated (M2) based on stimulatory environment. M1 macrophages degrade ECM and clear cell debris, and M2 macrophages promote angiogenesis and collagen deposition (Yang et al., 2021b). Further, M2b macrophages promote lymphangiogenesis to reduce myocardial fibrosis and cardiac dysfunction (Wang et al., 2022b). At the early stage following MI, macrophages and monocytes are almost exclusively derived from haematopoietic stem cells or extramedullary splenic reservoirs. They usually infiltrate the infarct area with pro-inflammatory phenotypes (Halade et al., 2018; Bajpai et al., 2019; Yap et al., 2023). Neonatally activated macrophages modulate angiogenesis, inflammation, and CMs proliferation, which is contrary to adult macrophages with different metabolites of oxygenation and nutrients (Lantz et al., 2021).

Herein, we describe the process of immune response after MI based on recent literature. At the beginning, these immune cells accumulate within hours, known as the inflammatory phase, and the peak recruitment of these immune cells occurs approximately 3 days after MI (Yap et al., 2023). Neutrophils are firstly recruited after MI and then phagocytized by proinflammatory CCR2+ Ly6C^high^ monocyte-derived macrophages with the replacement of some CCR2(-) resident macrophages, which increase anti-inflammatory and profibrotic cytokines (e.g., IL-10 and TGF-β) and decrease proinflammatory cytokines (e.g., IL-1β and TNFα) (Simões and Riley, 2022). Concurrently, macrophages phagocytize dead cells, and anti-inflammatory T cells (e.g., regulatory T cells/Tregs) are recruited (Dobaczewski et al., 2010; Hofmann and Frantz, 2015). After 1–2 days of MI, the anti-inflammatory phase (also known as proliferative phase) ensues, in which the macrophages undergo rapid proliferation with anti-inflammatory or reparative properties (Dick et al., 2019). At this stage, cytokines also promote CF-to-MF transformation. Briefly, the resolution of inflammation and reparative tissue remodeling are initiated. Further, the recovery phase follows after 3–7 days of MI, MFs and CCR2+ macrophages mediate fibrosis and scar formation (Yap et al., 2023). Moreover, the macrophages subsequently transform into reparative CCR2+ Ly6C^low^ macrophages *via* the transcriptional program dependent on a nuclear receptor subfamily four group A member 1, which promotes fibrotic scar formation *via* collagen deposition and MFs transformation (Simões and Riley, 2022). Additionally, histamine deficiency promotes monocyte/macrophage-to-MF transformation in MI-induced cardiac fibrosis (Zhu et al., 2022). In contrast, specifically marked Gata6(+) pericardial macrophages accumulate on the cardiac surface after MI and prevent fibrosis (Jin et al., 2022).

#### 2.4.2 Other immune cells

Due to the regulative functions of immunity, it presents an intriguing direction for therapeutic intervention about cardiac fibrosis. Therefore, it is very important to comprehensively understand phenotypes and behaviours of different immune cells after MI.

Currently, there are also the up-to-date knowledge about other immune cells, such as B cells, T cells, and eosinophils. For example, B cells infiltrate into damaged myocardium within 1–7 days (Adamo et al., 2020), and B cells deficiency downregulates cytokines (e.g., TNF-α, IL-1β, IL-6, and TGF-1β) and collagen synthesis to alleviate fibrosis after MI (Mo et al., 2021). Additionally, MMP-2 increases the cytotoxicity of CD8^+^ T cells in acute MI patients (Li et al., 2021g). Cross-priming dendritic cells activate cytotoxic CD8^+^ T cells to exacerbate inflammatory damage and fibrosis (Forte et al., 2021b). Moreover, OSU-ERb-012, an estrogen receptor-β agonist, inhibits CD4^+^ T cells and improves cardiac remodeling in the MI-induced HF mouse model (Rosenzweig et al., 2022). Tregs promote Ly6C^high^ monocyte conversion into M2 macrophages by secreting cytokines (e.g., IL-10, IL-13, and TGF-β) to initiate the anti-inflammatory or regenerative phase (Kino et al., 2020).

Furthermore, Tregs can directly regulate CFs (Kino et al., 2020). Eosinophils are increased in the blood and heart (mostly in the infarct area) in MI patients and mice, and eosinophil depletion promotes cardiac dysfunction and fibrosis (Liu et al., 2020a).

#### 2.4.3 Cytokines

Massive researches have demonstrated the role of cytokines in MI that cytokines not only form a complex network to regulate inflammatory response, but also can form cytokine storm to worse myocardium injure following cardiac decompensation (He et al., 2022). Upon cardiac injury, the inflammatory signaling molecules immediately increased.

DAMPs bind toll-like receptors (TLRs), activate inflammasomes (e.g., nod-like receptor protein 3 (NLRP3)), and promote cytokines/chemokines synthesis to induce activation and recruitment of immune cells and engage immune defenses (Schroder and Tschopp, 2010). Most commonly, IL-1 family includes pro-inflammatory and anti-inflammatory members. For example, IL-1α reduces the remodeling in border zone CFs by upregulating steroidogenic acute regulatory protein (Razin et al., 2021). Moreover, IL-33 mediates the shift in the inflammatory phase toward its resolution through IL-1R4 (Fearon and Fearon, 2008). MFs with physiological stretching release IL-33 to bind the ST-2 receptor on the CMs membrane to promote cell survival and integrity (Vianello et al., 2019), whereas IL-33 worsens cardiac remodeling by recruiting eosinophils (Ghali et al., 2020). In a myeloid IL-4 receptor-α deficiency model, insufficient fibrotic remodeling is induced *via* downregulated TIMPs and collagen I deposition (Song et al., 2021a; Song et al., 2021b). IL-21 induces apoptosis of Ly6C^low^ macrophages and prevents cardiac repair (Kubota et al., 2021). Furthermore, IL-38 influences dendritic cells to reduce inflammation and fibrosis (Wei et al., 2020).

#### 2.4.4 Chemokines

Extensive evidence also implicates chemokines in the pathogenesis of cardiac fibrosis. Chemokines are key regulators controlling the migration and positioning of immune cells, and various cells proliferation to promote structural remodeling and functional recovery of the heart with inflammation quickly subsiding (Frangogiannis, 2014; Ma, 2021). However, persistent cytokines induce late cardiac contractility and adverse outcome (Wang et al., 2018). In the affected myocardium and heart-draining lymph nodes, MI induces complementary B-cell responses, while B cells infiltrate the infarct zone *via* the CXC-motif chemokine ligand 13 (CXCL13, the ligand of CXCR5-CXC-chemokine receptor type 5 (CXCR5)) axis and induce TGF-β1 expression (Heinrichs et al., 2021). CXCL8 induces neutrophil infiltration, whereas CC chemokines, such as chemokine CC-motif ligand 2 (CCL2), mediate the recruitment of mononuclear cells (Chen and Frangogiannis, 2021). In addition, CCL2, also known as monocyte chemoattractant protein-1 (MCP-1), has a higher serum level in ST-segment elevation myocardial infarction (STEMI) patients, but lower plasma levels in MI patients without collateral circulation (Kobusiak-Prokopowicz et al., 2007; Sahinarslan et al., 2010). Furthermore, CXCL10 and CXCL12 have leukocyte-independent mediatory effects, directly modulating CFs (Chen and Frangogiannis, 2021). Additionally, single-cell sequencing has found different immune cell abundance (resting and activated mast cells, activated CD4 memory T cells) and high expression of chemokines in MI patients (CCL3, CXCL3, CXCL8, and CXCL16 in CD1C-CD141-dendritic cells and CCL4 and CCL5 in natural killer cells) (Zhou et al., 2022).

#### 2.4.5 Growth factors

To explore novel treatments targeting cardiac fibrosis, it is very important to identify and elucidate precise mechanisms of growth factors, such as PDGF, FGF, and TGF have been best studied.

The PDGF family is composed of cell division stimulators and has five subunits (PDGF-AA, PDGF-BB, PDGF-AB, PDGF-CC and PDGF-DD), as well as two receptors, PDGFRα and PDGFRβ. All PDGF members have been shown to play a role in cardiac fibrosis (Bertaud et al., 2023). For example, overexpression of PDGF-A modulates scar content, reduces scar size, and increases capillary and arteriolar density in the infarct border zone (Rashid et al., 2021), whereas PDGF-AB enhances angiogenesis and increases scar anisotropy (high fiber alignment) without affecting overall scar size or stiffness (Thavapalachandran et al., 2020).

The FGF family has 22 members and pleiotropic effects (Bertaud et al., 2023). For example, FGF21 inhibits inflammation and fibrosis by downregulating early growth response protein 1 (Li et al., 2021c; Li et al., 2021d). Additionally, FGF12 overexpression decreases collagen I and III and fibronectin in Ang II-induced CFs (Liu et al., 2022). FGF10 increases cardiomyocyte renewal and limits fibrosis to promote cardiac regeneration and repair, which suggests FGF10 may be a clinically relevant target for heart repair (Hubert et al., 2022). Conversely, FGF23 increases myocardial fibrosis and dysfunction *via* activating β-catenin and promoting the pro-fibrotic crosstalk between CMs and CFs in a paracrine manner (Hao et al., 2016; Leifheit-Nestler et al., 2018).

There are also other growth factors involved in cardiac fibrosis, such as neuregulin-1, a paracrine growth factor secreted by cardiac endothelial cells, modulates hypertrophic and fibrotic processes during early cardiac remodeling *via* the neuregulin-1/erythroblastic leukemia viral oncogene homolog (ERBB) four axis (Dugaucquier et al., 2020).

In this section, we mainly focus on the multifaceted contributions of diverse immune cells populations and mediators after MI. As immunity affecting the prognosis of MI patients has become an area of substantial therapeutic interest, we discuss novel interventions regarding immunity in a later section.

### 2.5 Molecular mechanisms

**FIGURE 1:**
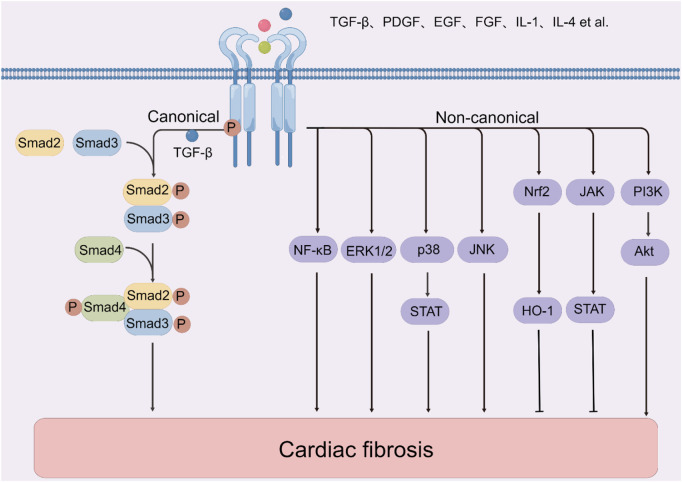
The molecular mechanisms of cardiac fibrosis. Note: Akt, protein kinase B; EGF, epidermal growth factor; ERK, extracellular signal-regulated kinase; FGF, fibroblast growth factor; HO-1, heme oxygenase-1; IL-1, interleukin-1; IL-4, interleukin-4; JAK, janus kinase; JNK, c-Jun N-terminal kinase; NF-κB, nuclear factor kappa-B; PDGF, platelet-derived growth factor; PI3K, phosphatidylinositol 3-kinase; STAT, signal transducer and activator of transcription 3; TGF-β, transforming growth factor β.

Many mechanisms (e.g., oxidative stress, inflammation, and mechanical stress) are known to affect cardiac fibrosis. Additionally, a large amount of antifibrotic therapy researches targeting underlying molecular mechanisms have been implemented with technological advancement (Figure 1) (The figure is drawn by figdraw). Due to the various signaling pathways involved in the process, an understanding of the precise mechanisms of cardiac fibrosis is still limited. From this viewpoint, it is essential to summarize and comprehend current evidence of cell signaling pathways associated with cardiac fibrosis. Hence, in the following sections, we summarize the recent data about the roles and crucial functions of several key signaling pathways in post-MI fibrosis: TGF-β, phosphatidylinositol 3-kinase (PI3K)/protein kinase B (Akt), nuclear factor erythroid 2-related factor 2 (Nrf2), mitogen-activated protein kinase (MAPK) and other molecular mechanisms. The molecularly targeted drugs may further decline the death rate of MI. Herein, it is indispensable and promising to target these aberrant signaling pathways and improve the pathological manifestations in post-MI fibrosis.

#### 2.5.1 TGF-β signaling pathway

TGF-β is the most typical cytokine regulating fibrosis and inducing collagen synthesis, which is secreted by macrophages into the ECM in an inactive form (pro-TGF-β) and then activated by proteases (e.g., plasmin, MMP-2, MMP-9, and ROS) (Takawale et al., 2015; Hata and Chen, 2016). Firstly, it binds TGF-β receptor(TGFβR)II, and then TGFβRI is phosphorylated and forms a receptor heterocomplex to react with smads protein. Bruton’s tyrosine kinase is up-regulated in MI, with directly binding and phosphorylating TGFβRI at tyrosine 182, and then activating the downstream to promote CF-to-MF transformation and the excessive ECM gene expression. And its second-generation inhibitor Acalabrutinib attenuates cardiac fibrosis (Wang et al., 2022a). Smads proteins have three types: receptor-regulated smad (smad1, smad2, smad3, smad5, and smad8), common smad (smad4), and inhibitory smad (smad6 and smad7). The TGF-β complex binds to R-smads (smad2 and smad3) and Co-smads (smad4), and then transfers into nucleus to regulate the transcription of target genes (Zhang et al., 2022c). The smad-dependent canonical pathway, coordinating with non-canonical pathways, transduces TGF-β signals (Zhang et al., 2022f).

TGF-β, as a critical molecule of MFs, promotes collagen synthesis, CF-to-MF transformation, and other fibrotic factors production, as well as activates multiple signaling pathways (Li et al., 2022b), while CFs and inflammatory cells express cystine knot protein gremlin-1 colocalizing with TGF-β and reduce collagen deposition (Müller et al., 2021). Further, TGF-β1 can activate CFs to increase collagen deposition, and sustained TGF-β1 expression subsequently leads to cardiac dysfunction (Xue et al., 2019). Moreover, TGF-β1 regulates ECM remodeling by promoting MMP/TIMP imbalance (Hodges et al., 2019). Additionally, a study has found that TGF-β3 expression gradually attained its peak in 1 month after MI with an opposite expression trend of TGF-β1 and TGF-β2 in MI patients, while TGF-β3 downregulated proliferation, migration, and collagen synthesis and upregulated lysyl oxidase and osteopontin in Ang II-induced human CFs and MI patients by promoting smad7 expression (Xue et al., 2019). The recombinant osteopontin activates cell-cycle re-entry in CMs, stimulates multiple cardiac cells, and improves scar formation, LV remodeling, and regional and global function after MI (Rotem et al., 2022).

Even if TGF-β2 and TGF-β3 are involved in cardiac fibrosis, the fibrotic effects triggered by the TGF-β family have primarily been attributed to TGF-β1 (Dewald et al., 2004). For example, phosphoglycerate mutase one deficiency suppresses inflammation, apoptosis, and fibrosis in post-MI by targeting TGF-β1 (Wu et al., 2021c). High serum tissue non-specific alkaline phosphatase (TNAP) level in MI patients can serve as a fibrotic biomarker and is positively correlated with mortality risk (Cheng et al., 2021), while TNAP inhibition provokes an antifibrotic effect through adenosine monophosphate-activated protein kinase (AMPK)/TGF-β1/smads and p53 (Gao et al., 2020b). Two inhibitory smads (smad6 and smad7) can prevent R-smad phosphorylation (Bertaud et al., 2023). Further, smad7 also restrains MFs activation by suppressing profibrotic ERBB2 in a TGF-β-independent manner (Humeres et al., 2022) but does not restrain the anti-inflammatory function of TGF-β in macrophages (Li et al., 2022d). Another member of the TGF-β superfamily, lefty1 alleviates post-MI CFs proliferation, differentiation, and secretion by suppressing the p-smad2 and p-ERK1/2 axis (Li et al., 2021a). Moreover, SH2 domain-containing protein tyrosine phosphatase-2 inhibits fibrosis *via* the ERK/smad pathway (Lu et al., 2021). Chordin-like one inhibits extracellular bone morphogenetic protein 4 (BMP4) to inhibit smad1/5/8 activation and autophagy in CMs and suppresses TGF-β1-induced fibrosis and CF-to-MF transformation (Ruozi et al., 2022).

Noteworthily, many non-coding miRNAs have been reported to be involved in cardiac fibrosis *via* regulating TGF-β signaling pathway (Zhao et al., 2022b). In this section, we summarize recent studies focusing on this pathway. Briefly, post-MI repair requires tight regulation of the TGF-β signaling pathway in case of excessive fibrosis and adverse remodeling leading to heart failure.

#### 2.5.2 PI3K/Akt signaling pathway

The PI3K/Akt/protein kinase B signaling pathway is one of the important intracellular signal transduction pathways. PI3K converts phosphatidylinositol 4,5-bisphosphate (PIP2) into phosphatidylinositol 3,4,5-trisphosphate (PIP3). Then PIP3 binds to the pleckstrin homology domain of Akt to alter its conformation and activate the downstream molecules, such as vascular endothelial growth factor (VEGF), endothelial nitric oxide synthase, while inhibiting mammalian target of rapamycin (mTOR) complex 1, glycogen synthase kinase 3β, forkhead box subfamily O, respectively (Zhang et al., 2022c). In recent years, basic research finds that targeting PI3K/Akt pathway is a beneficial signaling mechanism for anti-fibrotic treatments following AMI regulating cell proliferation, differentiation, migration and apoptosis. For example, Apelin-13 inhibits the PI3K/Akt axis to attenuate fibrosis in HF rats and AngII-induced CFs (Zhong et al., 2020). Further, visceral adipose tissue-derived serine protease inhibitor vaspin alleviates fibrotic remodeling and oxidative stress and decreases ANP, BNP, and collagen I and III by inhibiting the PI3K/Akt axis (Ji et al., 2022b). Additionally, inhibition of calcium and integrin binding protein one reduces cardiac fibrosis and levels of α-SMA, vimentin, and collagen I and III by inhibiting the PI3K/Akt pathway (Hu et al., 2022a). Since an essential requirement for the post-MI repair is recovering the capillary network in the injured area due to new vessel sprouting from existing ones (Gao et al., 2020a), transcription factor Yin-Yang one represses CMs apoptosis and fibrosis and promotes angiogenesis by enhancing Akt phosphorylation and increasing VEGF (Huang et al., 2021). The lysyl oxidase-like protein two increases MFs transformation, collagen fiber production and mechanical strength *via* the PI3K/Akt/mTOR pathway (Yang et al., 2016). By contrast, ivabradine prevents fibrosis and cardiac hypertrophy *via* suppressing PI3K/Akt/mTOR/p70S6K signaling (Yu et al., 2019; Dai et al., 2021). Klotho significantly reduces cardiac fibrosis and suppresses myocardial inflammation and apoptosis in MI-induced HF model *via* inducing autophagy through the inhibition of PI3k/Akt/mTOR signaling pathway (Wang et al., 2022d).

#### 2.5.3 Nrf2 signaling pathway

As a transcription factor and the product of the nuclear factor erythroid-derived 2-like 2 gene, Nrf2 consists of seven functional domains and participates in regulating oxidative stress and antioxidant genes (Ray et al., 2012; Hayes and Dinkova-Kostova, 2014). It transfers signaling molecules to the nucleus and initiates antioxidant gene transcription (Kensler et al., 2007). And its downstream target, heme oxygenase-1 (HO-1), is a rate-limiting enzyme that catalyzes heme to biliverdin Ixα, carbon monoxide, and iron (Wu et al., 2021a). Nrf2 signaling pathway plays a crucial role in post-MI remodeling. For example, in the MI rat model and Ang II-treated CFs, ghrelin ameliorates cardiac fibrosis by activating Nrf2 to inhibit the nicotinamide adenine dinucleotide phosphate (NADPH)/ROS pathway (Wang et al., 2021c; Wang et al., 2021d). In addition, Pinocembrin ameliorates cardiac remodeling by ROS clearance and Nrf2/HO-1 pathway activation, which further suppresses collagen fibers deposition and apoptosis and promotes angiogenesis (Chen et al., 2021d). Moreover, corosolic acid regulates the AMPK-α/Nrf2/HO-1 axis to inhibit cardiac fibrosis, oxidative stress, inflammation, and apoptosis (Wang et al., 2020i). Furthermore, Nrf2 reduces innate immune response in MI mice (Bromage et al., 2022). Thus, the protective effect of Nrf2/HO-1 following AMI should not be ignored. And it constitutes an appealing target for anti-fibrotic treatments. Plantarum activates Nrf2 antioxidant defense pathway and ameliorates cardiac dysfunction and collagen expression (Aboulgheit et al., 2021).

#### 2.5.4 MAPK signaling pathway

As a class of highly conserved serine/threonine protein kinases, MAPKs have four primary branches: ERK, c-jun N-terminal kinase (c-JNK), p38/MAPK and ERK5 (Gallo and Johnson, 2002; Cargnello and Roux, 2011). During variously physiological and pathological processes, these kinases can be sequentially activated and regulate proliferation, growth, and differentiation of cardiac cells, such as CMs, CFs, endothelial cells and macrophages (Muslin, 2008). In this section, we mainly introduce some recent studies of the MAPK pathway from the aspects of molecular regulation.

Calcium-activated chloride channels protein anoctamin-1 promotes CFs proliferation and secretion *via* the MAPK pathway (Tian et al., 2020). The upregulation of OUT domain-containing 7B suppresses phosphorylated focal adhesion kinase and ERK/p38 activities and reduces the levels of α-SMA and collagen I (Zhang et al., 2022b), while nicotinamide riboside kinase-2 regulates the p38 pathway to alleviate post-MI scar size and fibrosis (Ahmad et al., 2020). Further, zinc finger protein ZBTB20 protects the heart by inhibiting the JNK pathway (Li et al., 2020a; Li et al., 2020b), while melatonin improves myocardial fibrosis in the infarct border zone and apoptosis *via* the JNK/p53 pathway after MI in a diabetic mouse model (Lu et al., 2020). In oxygen-glucose deprivation/reoxygenation (OGD/R)-induced H9c2 cells and myocardial fibrosis model of mice, protocatechualdehyde, a major component from Salvia miltiorrhiza, against ischemic injury by suppressing endoplasmic reticulum stress-associated protein kinase R-like endoplasmic reticulum kinase/transcription factor 6α/inositol-requiring enzyme1α pathways (Wan et al., 2021).

In short, comprehensive study and understanding the mechanism of the MAPK pathway, taking this signaling pathway as the anti-fibrotic target are the keys to address challenges of post-MI fibrosis.

#### 2.5.5 Other molecular mechanisms

In addition to the above signaling pathways, other pathways have also been shown to be related to cardiac fibrosis. For example, ELABELA peptide increases angiogenesis and reduces cardiac interstitial fibrosis through activating ERK/hypoxia-inducible factor-1alpha/VEGF pathway in MIRI rat model (Rakhshan et al., 2022). A transcriptional complex (A-kinase anchoring protein 2, protein kinase A, and steroid receptor coactivator 3) modulates proangiogenic and antiapoptotic processes *via* protein kinase A-mediated phosphorylation and estrogen receptor α activation (Maric et al., 2021).

As a family of signal-dependent transcription factors, nuclear factor kappa B (NF-κB) is located in the cytoplasm in an inactive form, but it migrates to the nucleus following stimulation, and regulates its targets *via* binding to NF-κB response elements on the DNA (Li and Verma, 2002). As a typical pro-inflammatory signaling pathway, NF-κB regulates gene transcription and promotes inflammatory responses (He et al., 2022), for example, exendin-4 regulates the NF-κB axis to prevent inflammation and cardiac remodeling (Eid et al., 2020), and Nur77 improves cardiac fibrosis by inhibiting the NF-κB-dependent pathway (Chen et al., 2021a). Further, hippo pathways are vital mechanisms of cardiac repair. For example, hippo pathway kinases Lats1/2 inhibit yes associated protein (YAP)-induced injury response, while conditional deletion of Lats1/2 in adult resting CFs initiates CF-to-MF transformation (Xiao et al., 2019). Moreover, platelet-activating factor receptor and YAP1 are significantly increased in MI mice, accompanying with its positive feedback loop in cardiac fibrosis (Li et al., 2022f).

In addition to the common signaling pathways noted above, other recent studies of molecular mechanisms are summarized. For example, researchers have found that fibrosis is associated with calmodulin/p38/signal transducer and activator of transcription (STAT) 3, wnt/β-catenin, TLR4/calmodulin-dependent protein kinase II and B lymphoma Mo-MLV insertion region 1 homolog/p15/retinoblastoma pathways et al. (Li et al., 2020g; Han et al., 2020; Yang et al., 2021c; Fu et al., 2021; Bugg et al., 2022; Zhang et al., 2022e; Shi et al., 2022).

Crosstalk also exists in different signaling pathways; for instance, endogenous TGF-β1 repressor SKI activates the hippo pathway *via* LIM domain-containing protein one to inhibit CFs activation (Landry et al., 2021). There are some studies about ion channel; for instance, the mechanosensitive ion channel transient receptor potential vanilloid four deletion regulates CF-to-MF transformation to improve harmful remodeling after MI (Adapala et al., 2020; Adapala et al., 2021). Piezo1 activates CFs to induce MFs recruitment and excessive ECM deposit (Braidotti et al., 2022), while coronary vascular endothelial sodium channel activation promotes cardiac fibrosis and dysfunction (Hill et al., 2022). Further, embryonic CFs of mice with mitochondrial Ca^2+^ uniporter deletion are more sensitive to Ca^2+^ overload than normal CFs (Huo et al., 2020). Briefly, it is necessary to comprehensively understand the molecular mechanisms of cardiac fibrosis after MI before performing specific interventions.

To sum up, it is critical to develop and optimize therapeutic strategies according to fundamental mechanisms and pathophysiology of cardiac fibrosis. To date, research in basic science has disclosed a range of pathophysiological mechanisms of post-MI cardiac fibrosis, and many attractive inhibitors and antagonists have been developed based on the molecular mechanisms. However, a lot of them have not currently been launched in human clinical trials to advance toward clinical application, and investigations remain challenging and need to be studied further.

## 3 Evaluations of cardiac fibrosis

### 3.1 Cardiac magnetic resonance late gadolinium enhancement

Cardiac magnetic resonance (CMR) uses different sequences and modalities to assess the heart, such as extracellular volume, derived from T1-weighted imaging, examination of total interstitial space, T2 mapping and weighted imaging examination of edema, and late gadolinium enhancement (LGE) examination of scar and fibrosis (Kali et al., 2014; Gupta et al., 2021). Because extracellular space is enlarged by dead CMs and post-infarct fibrosis, CMR-LGE imaging with excessively retained gadolinium-based contrast agents represents a non-invasive standard for assessing myocardial viability and fibrosis (Holtackers et al., 2022); for instance, a subendocardial scar can be detected *via* dark-blood LGE-CMR (Holtackers et al., 2021). Feature tracking of CMR accurately quantifies cardiac strain, and a retrospective study has found that almost 75% of acute scars and 80% of subacute scars could be detected by CMR with a segmental circumferential strain of native cine sequences (Polacin et al., 2022). In the mouse MI models with monocyte populations deletion, elastin deposition, as an inflammatory response and a potential fibrotic biomarker, could be detected *via* CMR with an elastin/tropoelastin-specific contrast agent (Elkenhans et al., 2021). However, the use of CMR is limited by availability, time, cost, and severe renal insufficiency as an adverse effect of contrast administration, which can be solved by segmental peak circumferential strain calculation (Polacin et al., 2022). Additionally, CMR screens post-MI patients at risk of ventricular tachycardia by identifying and quantifying a heterogeneous scar zone and substrate features (Merino-Caviedes et al., 2021). A case-control study has retrospectively reviewed LGE-CMR data of chronic post-MI and found that border zone channel mass was the strongest independent scar-derived variable and precision risk stratification relevant to sustained monomorphic ventricular tachycardia, while border zone channel mass was associated with the qualitative structure, heterogeneity, spatial distribution, and slow conducting channels within the scar (Jáuregui et al., 2022).

### 3.2 Echocardiography

Early detection of myocardial fibrosis can identify patients at risk of adverse events, which is independently associated with a measure of scar between echocardiography and defibrillator intervention (Gaibazzi et al., 2020). A large single-center clinical cohort study has found that diastolic dysfunction effectively identified mortality risk with relevance to higher incidence and extent of scar *via* echocardiography and LGE (Wang et al., 2020a; Wang et al., 2020b; Wang et al., 2020c; Wang et al., 2020d). Speckle tracking echocardiography (STE) can evaluate MI *via* end-systolic radial strain peak to reflect segmental scar with very high sensitivity and specificity, and when combined with blood pressure, non-invasive myocardial work parameters (e.g., myocardial work index, constructive work, and myocardial work efficiency) can be obtained and are significantly lower in the segment with the largest LGE than without LGE after contrasting with gadolinium (Mahdiui et al., 2021). These parameters are emerging potential markers of segmental myocardial viability, prognostic markers, and therapeutic targets in STEMI patients with primary percutaneous coronary intervention (PCI) (Mahdiui et al., 2021). Moreover, LV mechanical dispersion measured by CMR and STE is correlated with scar burden as a prognostic parameter (Holtackers et al., 2021).

### 3.3 Computed tomography

Cardiac computed tomography (CT) estimates the extracellular volume and macroscopic scar *via* CT delayed enhancement (CT-DE) with relatively low iodine contrast compared to CMR-LGE (Gupta et al., 2021). X-ray microCT implements quantitative 3D analysis and visualization of cardiac fibrosis in MI mice (Janbandhu et al., 2022).

### 3.4 Molecular imaging

CFs activation is promising for targeted therapy, which can be detected and tracked by fibroblast activation protein (FAP) imaging with novel radiotracer [68Ga]MHLL1 (Langer et al., 2021). FAP imaging has also detected activated CFs in the non-edematous and non-infarcted area in a prospective study of reperfused STEMI patients (Xie et al., 2022). Moreover, FAP-α deletion attenuates cardiac dilation in MI mice (Hoffmann et al., 2021). Additionally, a retrospective study has found a strong correlation between CFs activation volume with cardiac function and peak creatine kinase *via* 68Ga-FAP-α inhibitor positron-emission tomography (PET) (Kessler et al., 2021), while PET and single-photon emission computed tomography (SPECT) indirectly assessed cardiac fibrosis *via* myocardial perfusion imaging (Gupta et al., 2021). PET immunoimaging DOTATATE tracers can find high expression of somatostatin receptor two in M1 inflammatory macrophages (Toner et al., 2022). Multiparametric imaging characterizes the immune response transforming to tissue repair after MI (Hess et al., 2022).

### 3.5 Biomarkers

Rapidly advancing technologies favor post-MI fibrotic biomarkers identification, such as MMP, collagen peptides, galectin-3, and ST-2 (Bostan et al., 2020), which can be combined with MI biomarkers, such as creatine kinase-myocardial band (CK-MB), troponin, and N-terminal-pro type brain natriuretic peptides (NT-proBNP). A prospective study of 92 patients over 70 years old with MI finds that patients over 70 years old with MI and fragility have significantly higher levels of myocardial stress and fibrosis (Aidumova et al., 2022). Galectin-3, a β-galactoside-binding lectin mainly synthesized by macrophages, maintains cardiac structure and function in the early MI stage, and promotes tissue fibrosis and scar formation in the late stage (Leancă et al., 2022). The TNF-α, soluble tumour necrosis factor-α receptor-1 and 2 and oxidative stress could be considered as potential non-invasive diagnostic and therapeutic biomarkers for coronary chronic total occlusion in the oldest patients with coronary heart disease (Li et al., 2020e). Additionally, low miR-26a plasma level is highly correlated with certain markers (e.g., CK-MB and troponin I) in STEMI patients (Chiang et al., 2020), and immunoreactivity of Nε-(carboxymethyl)lysine is positively correlated with NT-proBNP and cardiac fibrosis (Nogami et al., 2020). Furthermore, abnormal myocardial collagen I and III release certain peptides in the circulation as fibrotic markers (Nikolov and Popovski, 2022), such as PICP and PIIINP, directly correlating with indexes of cardiac diastolic function (Osokina et al., 2020). Furthermore, serum PIIINP of ≥381.4 ng/ml on the 12th day increases the risk of cardiac fibrosis 1 year after the disease onset in STEMI patients with preserved ejection fraction (EF) of I–III degree (Osokina et al., 2021). Moreover, human epididymis factor-4 is an independent predictor of low EF as a diagnostic marker and therapeutic target in cardiac fibrosis (Kilci et al., 2021). ST-segment resolution (STR) is a marker for severe myocardial fibrosis and is associated with scar thickness and size, while STEMI patients with STR of <40.15% easily develop transmural scars (Dong et al., 2021b). The combination of the ICTP/PIIINP ratio and ST2 might aid in risk stratification and serve as prognosis biomarkers in HF patients (Dupuy et al., 2019).

In addition to the above, more and more new technologies have emerged, such as Bayesian cardiac strain imaging assessing murine cardiac fibrosis (Al Mukaddim et al., 2022), single-cell mRNA sequencing inspecting dynamic interstitial cell response in MI mice (Forte et al., 2020), stereological method quantifying CMs (Mühlfeld and Schipke, 2022), and high-throughput screening differential genes expression of monocytes-CFs communication (Wu et al., 2022b; Wu et al., 2022d).

Therefore, developing new tools that allow both an early detection of cardiac fibrosis and the determination of its origin and characteristics will potentially lead to the rapid and efficient treatment of patients.

## 4 Interventions for cardiac fibrosis

**FIGURE 2:**
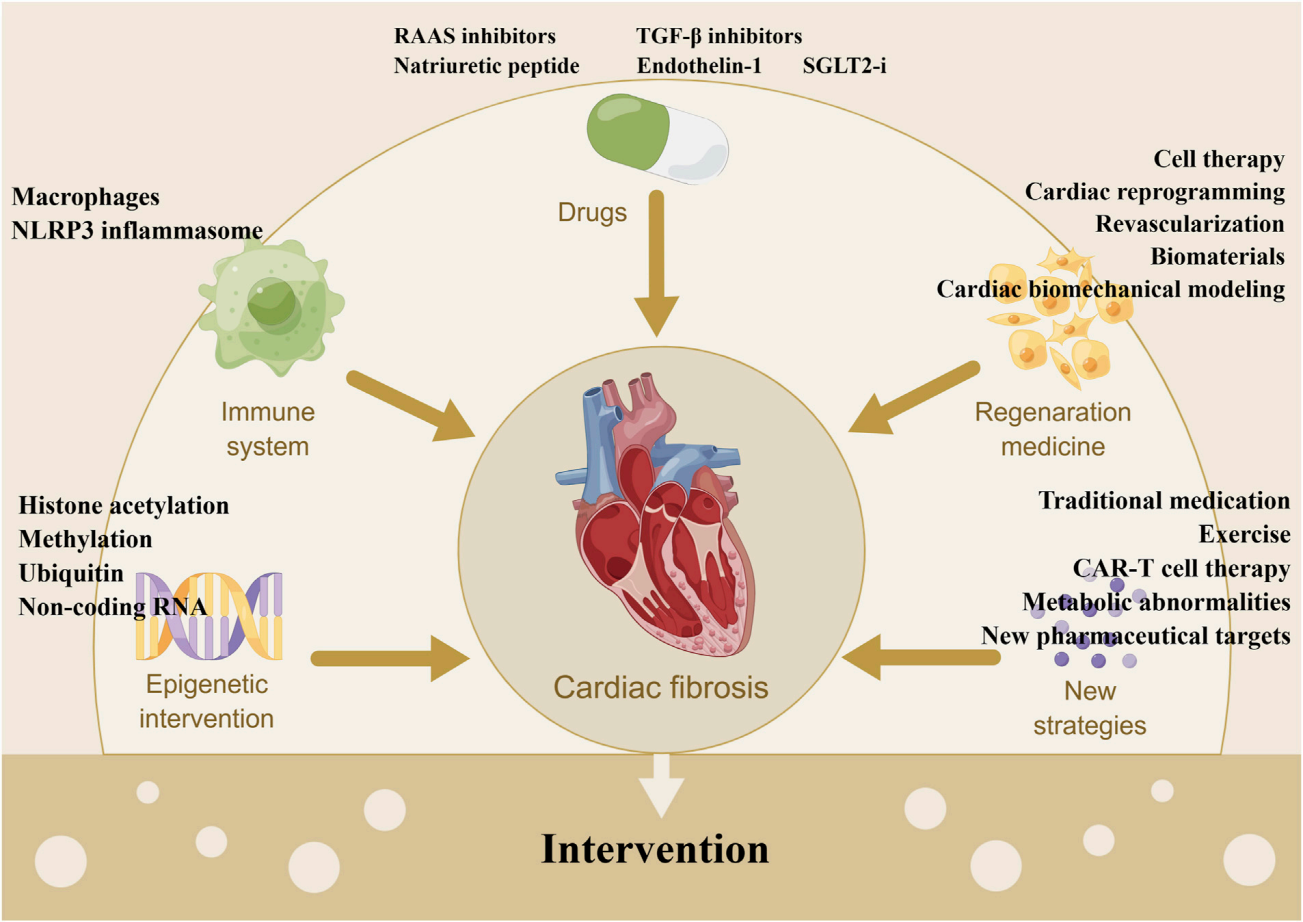
Summary of the interventions of cardiac fibrosis. Note: CAR, chimeric antigen receptor; NLRP3: nod-like receptor protein three; RAAS: renin-angiotensin-aldosterone system; SGLT2-i: sodium-glucose cotransporter-2 inhibitor; TGF-β: transforming growth factor β.

The therapies against cardiac fibrosis are still a focus of clinical attention, and how to reverse fibrosis is always a hot topic. Unfortunately, the effective measures are still lacking and lead to the devastating clinical outcomes, despite the various encouraging results from experimental studies (Morfino et al., 2022). In the studies of clinical drugs, RAAS antagonists have been shown to attenuate cardiac fibrosis and dysfunction, with clinical applications limited by their hypotensive effects and inability to stop the fibrotic progression (AlQudah et al., 2020). Conversely, TGF-β inhibitors (e.g., pirfenidone) improve fibrosis, without affecting blood pressure, but with unexpected side effects (e.g., liver toxicity). At the meantime, some known drugs are going through different phases of clinical trials (Table 1), such as RAAS inhibitors, sodium-glucose cotransporter-2 inhibitors (SGLT2is), BNP, and GRK2 inhibitors**,** in forms of monotherapy alone or combined with other drugs. Furthermore, fibrosis involves multiple molecules and processes (e.g., inflammatory cells recruitment, molecular mediators release, collagen synthesis, cells differention), which suggests small molecules targeting fibrosis would be the promising interventions. However, these novel therapies are still limited in preclinical studies without the validation of clinical efficacies against fibrosis. For the development of anti-fibrotic drugs, it is great important to apply novel molecular targets or drug repurposing *via* screening drugs tested and approved for other indications. In this section, we focus on the known drugs, novel compounds and other treatments with anti-fibrotic effects, which are shown in Figure 2 (The figure is drawn by figdraw).

**TABLE 1:** Recent progress in clinical trials for treating fibrosis and its complications.

	Interventions	NCT number	Status	Phases	Included patients (n)	Research topic
**Renin inhibitor**	Aliskiren	NCT00414609	Completed	Phase 3	820	Safety and efficacy of Aliskiren in patients with MI
**ACEI and ARB**	Ramipril, Candesartan Cilexetil, Allopurinol	NCT01052272	Completed	Phase 2|Phase 3	72	Impact of diabetes on left ventricular remodeling
	Ramipril, Irbesartan	NCT00517322	Unknown status	Phase 4	80	Left atrial remodeling in hypertension: effects of Ramipril or Irbesartan
	Lisinopril	NCT03422705	Not yet recruiting	Phase 2	75	Preventing adverse remodeling following pacemaker implantation
	Telmisartan, Amlodipine	NCT03956823	Unknown status	Not Applicable	300	Clinical efficacy of Telmisartan in reducing cardiac remodeling among obese patients with hypertension
	Candesartan, Diltiazem, Bisoprolol	NCT01162902	Unknown status	Phase 4	150	Comparison of vascular remodeling between different antianginal medication
	Losartan	NCT05607017	Not yet recruiting	Early Phase 1	10	Losartan in prevention of radiation-induced HF
	Valsartan	NCT00133328	Completed	Phase 4	3000	A morbidity-mortality and remodeling study with Valsartan
	Valsartan	NCT01340326	Completed	Phase 4	800	The impact of dose of Valsartan and genetic polymorphism on ventricular remodeling after MI
**ARNI**	Sacubitril/Valsartan	NCT02887183	Completed	Phase 4	794	Effects of Sacubitril/Valsartan therapy on biomarkers, myocardial remodeling and outcomes
	Sacubitril/Valsartan, Valsartan	NCT03552575	Completed	Phase 3	93	Effects of Sacubitril/Valsartan vs. Valsartan on left ventricular remodeling after MI
	Sacubitril/Valsartan, Amlodipine	NCT04929600	Recruiting	Phase 4	120	Sacubitril/Valsartan *versus* Amlodipine in hypertension and left ventricular hypertrophy
	Sacubitril/valsartan	NCT05089539	Not yet recruiting	Phase 2	60	The Effect of Sacubitril/Valsartan on cardiac fibrosis in patients with HFpEF
	Sacubitril/Valsartan, Enalapril, Valsartan	NCT04912167	Not yet recruiting	Phase 3	376	The Effects of Sacubitril-Valsartan vs. Enalapril on left ventricular remodeling in STEMI
**Adrenergic receptor inhibitors**	Seloken ZOK/Toprol-XL	NCT00038077	Completed	Phase 3	300	Reversal of ventricular remodeling with Toprol-XL
	Carvedilol, Metoprolol succinate, Metoprolol succinate + doxazosin	NCT01798992	Completed	Phase 4	56	Effect of Beta-blockers on structural remodeling and gene expression
**MRA**	Eplerenone	NCT00082589	Completed	Phase 4	250	Effect of Eplerenone in patients with mild to moderate HF
	Eplerenone	NCT00132093	Completed	Phase 4	100	Effects of Eplerenone on left ventricular remodeling following heart attack
	Spironolactone	NCT01069510	Completed	Phase 2	40	Spironolactone in adult congenital heart disease
**Other diuretics**	Torasemide, Furosemide	NCT00409942	Completed	Phase 4	142	Effect of a new formulation of Torasemide on myocardial fibrosis in patients with HF
	Furosemide	NCT04628325	Completed	Phase 3	136	Effects of Furosemide plus small HSS in subjects with HFrEF
**Inflammation modulators**	Colchicine	NCT03156816	Completed	Phase 2	194	Colchicine for left ventricular remodeling treatment in AMI
	Colchicine	NCT05709509	Recruiting	Phase 4	148	Effect of Colchicine on MMP-9, NOX2, and TGF-β1 in MI
	Anakinra	NCT01175018	Completed	Phase 2	30	Anakinra to prevent adverse post-infarction remodeling
**TGF-β inhibitor**	Pirfenidone	NCT02932566	Completed	Phase 2	129	The Efficacy and safety of Pirfenidone in patients with HFpEF
**Prostacyclin analogs**	Beraprost	NCT05103813	Recruiting	Early Phase 1	100	Effect of Beraprost on reperfusion therapy for acute STEMI
	beprostaglandin	NCT05043558	Not yet recruiting	Phase 1|Phase 2	220	Effect of Prostaglandin on coronary microcirculation and ventricular remodeling after reperfusion therapy in acute STEMI
**Platelet aggregation inhibitors**	Ticagrelor, Clopidogrel	NCT02224534	Unknown status	Phase 4	326	Ticagrelor *versus* Clopidogrel in left ventricular remodeling after STEMI
**MMPs**	Doxycycline	NCT00469261	Completed	Phase 2	110	Doxycycline and post myocardial infarction remodeling
	Doxycycline	NCT03960411	Unknown status	Phase 3	80	Effect of Doxycycline on cardiac remodeling in STEMI patients
**BNP**	BNP	NCT04033861	Unknown status	Phase 4	352	Early rhBNP on myocardial remodeling and reperfusion in patients with STEMI
**SGLT2i**	Dapagliflozin	NCT02397421	Completed	Phase 4	56	Safety and effectiveness of SGLT2i in patients with HF and DM
	Dapagliflozin	NCT03782259	Completed	Phase 4	60	Effects of SGLT2i on myocardial fibrosis and inflammation in patients With DM2
	Dapagliflozin	NCT05606718	Recruiting	Phase 4	98	Effect of Dapagliflozin on functional mitral regurgitation and myocardial fibrosis
	Dapagliflozin	NCT04783870	Recruiting	Phase 4	60	Effect of Dapagliflozin on left ventricular remodeling after AMI
	Dapagliflozin	NCT05305911	Recruiting	Phase 2	80	SGLT2i and STEMI
	Empagliflozin	NCT04461041	Recruiting	Phase 4	164	Effect of Empagliflozin on cardiac remodeling in people without DM
	Ertugliflozin	NCT04490681	Unknown status	Phase 3	52	Validation of Ertugliflozin for inhibiting cardiac fibrosis in heart failure patients with non-ischemic cardiomyopathy
**Antilipemic agents**	Rosuvastatin	NCT00240292	Completed	Phase 3	160	Effect of Rosuvastatin on ventricular remodeling lipids and cytokines
	Rosuvastatin	NCT00505154	Completed	Phase 3	75	Effect of Rosuvastatin on left ventricular remodeling
	Rosuvastatin, Evolocumab	NCT05613426	Not yet recruiting	Phase 4	330	Effect of evolocumab on left ventricular remodeling in patients with anterior STEMI undergoing primary PCI
	Atorvastatin	NCT00795912	Completed	Phase 4	56	Effect of Statins in patients with HF
	Atorvastatin	NCT00286312	Completed	Phase 4	50	Effect of Atorvastatin on MI size
**GRK2 Inhibitors**	Paroxetine	NCT03274752	Completed	Phase 2	50	Paroxetine-mediated GRK2 inhibition to reduce cardiac remodeling after AMI
**Others**	Regadenoson	NCT02589977	Completed	Phase 4	55	Evaluation of myocardial blood flow, interstitial fibrosis and oxidative metabolism in HFpEF
	Epoetin alfa	NCT00378352	Completed	Phase 2	223	Effect of erythropoietin on ventricular remodeling in patients with AMI
	Calcifediol	NCT02548364	Active, not recruiting	Phase 3	109	Effect of Vitamin D on ventricular remodeling in patients with AMI
	Ivabradine	NCT05348057	Recruiting	Phase 4	240	Effect of Ivabradine on the improvement of left ventricular remodeling in STEMI patients after primary PCI

Note: ACEI, angiotensin-converting enzyme inhibitors; ARB, angiotensin receptor blockers; BNP, brain natriuretic peptide; GRK, G-protein-coupled receptor kinase; HF, heart failure; HFpEF, heart failure with preserved ejection fraction; HFrEF, heart failure with reduced ejection fraction; HSS, hypertonic saline solutions; MI, myocardial infarction; MMPs, matrix metalloproteinases; MRA, mineralocorticoid receptor antagonists; NOX2, nicotinamide adenine dinucleotide phosphate oxidase 2; PCI, percutaneous coronary intervention; RAAS, Renin-Angiotensin-Aldosterone System; SGLT2i, sodium-glucose cotransporter 2 inhibitor; STEMI, ST-segment elevation myocardial infarction; TGF-β, transforming growth factor β.

### 4.1 Pharmaceutical interventions

#### 4.1.1 RAAS inhibitors

Several clinical trials of RAAS inhibitors are currently progressing in different phases, which are presented in Table 1. Other non-clinical trials have been reported in the recent years. For example, sliskiren is not only the first Food and Drug Administration-approved and orally active renin inhibitor to treat hypertension, but also regulates collagen metabolism and cardiac fibrosis *in vivo* and *in vitro* (Kleinert, 1996; Zhi et al., 2013). Additionally, compound 21 is a non-peptide AT2R agonist with antifibrotic effect (Wang et al., 2017). Targeting RAAS represents a promising therapeutic approach to combat fibrosis, however, conventional RAAS inhibitors cannot completely hamper the fibrotic progression. Together with ACEI, ARNI sacubitril/valsartan suppresses cardiac dysfunction and fibrosis *via* the downregulation of TGF-β1, BNP, α-SMA, vimentin (Liu et al., 2021b). Furthermore, valsartan and sacubitril/valsartan prevent adverse remodeling in MI rats by reducing oxidative stress, inflammation, and fibrosis (Raj et al., 2021). In MI with hypertensive rats model, the mineralocorticoid receptor antagonist (MRA) spironolactone reduces CFs, MFs, and macrophages infiltration in the heart and kidney (Leader et al., 2021); however, it also binds other steroid receptors (e.g., progesterone and androgen receptors) causing side effects (e.g., gynecomastia and galactorrhea) (Weldon and Brown, 2019). Moreover, eplerenone, a well-tolerated selective MRA, decreases PIIINP with good efficacy when baseline PIIINP is ≥3.6 mmol/L after MI with HF or diabetes (Stienen et al., 2020). As a centrally acting aminopeptidase A inhibitor prodrug, QGC606 inhibits the overactivation of the brain renin-angiotensin system and fibrotic remodeling without lowering blood pressure (Boitard et al., 2022).

#### 4.1.2 TGF-β inhibitors

Antifibrotic drug pirfenidone, a TGF-β1 inhibitor, has been identified from molecular information and transcriptomic data in a swine MI model (Aimo et al., 2022a), whereas evidence in humans has been limited to a phase 2 study evaluating extracellular volume changes with CMR (Aimo et al., 2022b). The clinical use of pirfenidone is limited by the high doses and various side effects. In an article, the total releasing duration of pirfenidone is prolonged by using acellular peritoneal matrix-loaded pirfenidone nanodroplets, which alleviates cardiac fibrosis (Fu et al., 2022b). Except pirfenidone, there are several interventions targeting TGF-β signaling pathway against cardiac fibrosis, such as dihydrolycorine, choline, indole alkaloids, and indole derivatives (Qin et al., 2022). Moreover, 2,5-dimethylcelecoxib inhibits the TGF-β axis and suppresses CF-to-MF transformation in a cryoinjury-induced MI model (Ikushima et al., 2022). The caffeic acid p-nitro phenethyl ester-pNO2 suppresses fibrosis, inflammation, and apoptosis *via* the TGF-β1/Gal-3 pathway (Wan et al., 2022b). Additionally, thymosin β4 decreases MFs growth and TGF-β1-induced activation to reduce fibrosis (Wang et al., 2022c). Salinomycin inhibits CFs activation and ECM secretion *via* the inhibition of TGF-β1-dependent p38/MAPK and Rho-kinase pathway in CFs Ang II-infused mice (Burke et al., 2021b). Nintedanib, another antifibrotic agent, was approved to improve pulmonary fibrosis. But the evidence for its role in the treatment of cardiac fibrosis is still lacking (Yvette, 2021).

#### 4.1.3 Drugs targeting the natriuretic peptide family

Strong evidence shows that NPs treatment has beneficial effects on post-MI cardiac remodeling. For example, a previous study has demonstrated that intravenous administration of ANP inhibited RAAS, SNS activity, MIRI, and cardiac remodeling in MI patients (Kasama et al., 2008). Another study has found that continuous CNP infusion (0.1 mg/kg/min) by osmotic mini-pump for 2 weeks after permanent coronary artery occlusion prevents cardiac remodeling (Wang et al., 2007). Further, when BNP (15 mg/kg/day) is intravenously injected over 8 weeks in rats with permanent coronary occlusion, BNP treatment prevents cardiac hypertrophy and EF decline and decreases plasma Ang II level and collagen content in the myocardium (He et al., 2009). Furthermore, after the genetic knockout of *Npr1* gene encoding NPR-A in mice, blood pressure rises, and cardiac hypertrophy develops. Blood pressure becomes elevated by 41 mmHg in *Npr1* ^−/−^ mice, together with a 60% increased heart weight/body weight ratio and CM hypertrophy. These findings indicate that endogenous NPs can prevent the development of cardiac hypertrophy (Vellaichamy et al., 2014; Pandey, 2018). A fourth phase clinical trial is ongoing to evaluate early rhBNP intervention in myocardial remodeling and reperfusion in patients with STEMI (Table 1). However, the current evidence mainly exists at the level of animal experiments, and future clinical applications still need to be further explored.

#### 4.1.4 Endothelin-1

ET-1 plays a major role in regulating myocardial fibrosis in several pathological conditions, and its receptor blocker might be beneficial in attenuating biventricular remodeling (Ramos et al., 2018). A few decades ago, studies found that ET-1 A and ET-1 A/B receptor antagonists substantially improved survival, cardiac function, and adverse cardiac remodeling (Sakai et al., 1996; Fraccarollo et al., 2002). Current evidence suggests that ET-1 is not only a molecular marker of cardiac fibrosis but also a novel therapeutic target (Hoffman et al., 2019). However, there is still no clinical evidence that drugs such as bosentan and enversentan have therapeutic effects on cardiac fibrosis.

#### 4.1.5 Sodium-glucose cotransporter-2 inhibitors

In experimental studies and clinical trials, it has been demonstrated that SGLT2is are cardioprotective independently from controlling glucose, for instance, canagliflozin attenuates fibrosis *via* reducing JAK/STAT signaling, activating adenosine monophosphate-activated protein kinase, and antioxidant signaling (Sabe et al., 2023). In clinical practice, empagliflozin can significantly reduce mortality and hospitalization of HF patients (Kang et al., 2020). It reduces collagen deposit and fibrosis without improving cardiac function *via* the inhibition of the TGF-β1/smad3 pathway during the early post-MI period (Daud et al., 2021). However, another study reports that the short-term and low-dose empagliflozin increases cardiac systolic function by downregulating MMP-9 and sodium hydrogen exchanger one and upregulating sarco/endoplasmic reticulum Ca^2+^-ATPase without changing arterial stiffness, blood pressure, fibrotic markers levels, and necroptosis (Goerg et al., 2021). Another selective SGLT1 inhibitor KGA-2727 improves fibrotic remodeling in MI mice (Sawa et al., 2020). Moreover, the DELIVER trial finds dapagliflozin reduces the combined risk of worsening heart failure or cardiovascular death among patients with mildly HFpEF (Solomon et al., 2022). In HFpEF pigs, it also decreases hypertension and reverses concentric remodeling of the heart, with the inhibition of inflammatory response and NO-cGMP-PKG pathway activation (Zhang et al., 2019b). Regrettably, SGLT2i cannot reduce extracellular volume expansion expanded by myocardial interstitial fibrosis (Bojer et al., 2022). Except SGLT2i, there are also other hypoglycemic agents associated with cardiac fibrosis. For example, metformin reduces collagen IIIA1, α-SMA, and CD68 levels after 2 weeks of reperfusion and improves fibrotic remodeling (Loi et al., 2021). Furthermore, metformin and cyclosporin A exert cardiac protection by regulating the balance between AMPK and apoptosis in the mitochondria of bile duct-ligated rats (Moheimani et al., 2021).

### 4.2 Targeting the immune system

The immune system is activated by MI, which can be a therapeutic target. Thus, immune-related interventions have been a study hotspot with the advance of precision medicine; for instance, the injection of human vascular cell adhesion molecule 1-expressing CFs restores cardiac function by promoting lymphangiogenesis (Iwamiya et al., 2020). Additionally, CD34 cells, isolated from mobilized human mononuclear peripheral blood cells, reduce cardiac scar and fibrosis in MI mice (Tripathi et al., 2020). A ligand-binding blocking anti-CD28 monoclonal antibody improves post-MI healing in mice (Gladow et al., 2020). Furthermore, the immune checkpoint programmed cell death protein one inhibits immune response to prevent damage, and its depletion increases T-cell infiltration in reperfused MI (Michel et al., 2022). In addition, high B cell counts are correlated with enhanced EF in MI patients with PCI, and empagliflozin can treat the MI-induced B cell developmental arrest (Xu et al., 2022b). Moreover, glucocorticoids released by the neuroendocrine system induce Na^+^/H^+^-exchanger 1-mediated autophagic death of bone marrow B cells and reduce B cell progenitor proliferation and differentiation (Xu et al., 2022b). Another study reports that adoptive transfer of atorvastatin-induced tolerogenic dendritic cells alleviates CMs apoptosis, fibrosis, inflammatory cells infiltration, and oxidative stress by suppressing TLR-4/NF-κB pathway in MI (Wang et al., 2023).

#### 4.2.1 Targeting the macrophages

Recently, massive studies focus on the detrimental macrophages as the antifibrotic therapeutic targets. For example, cardiac rupture may be induced by macrophage-induced inflammation and downregulated activation of reparative MFs. In the macrophage protease-activated receptor two knockout mice, there are down-regulation of proinflammatory cytokines, recruitment of macrophages, fibrosis in a remote area, and macrophage-derived interferon-β expression, which stimulate the JAK/STAT3 pathway in CFs (Zuo et al., 2020). In addition, granulocyte colony-stimulating factor (G-CSF) improves cardiac remodeling by upregulating JAK2/STAT3 axis (Wang et al., 2020h). Further, N-Propargyl caffeate amide promotes pro-resolving macrophage polarization and prevents cardiac fibrosis by activating peroxisome proliferator-activated receptors-γ (PPAR-γ) pathway (Cheng et al., 2020). Interestingly, cortical bone stem cells can induce a novel macrophage phenotype to modify cardiac inflammation after MI (Hobby et al., 2021). Moreover, hypoxia-induced mitogenic factor deletion promotes M2 macrophages and inhibits M1 macrophages polarization to improve cardiac repair (Li et al., 2021f). M2b macrophages reduce the amount of collagen I and α-SMA, proliferation and migration of CFs, and differentiation of CFs into MFs, whereas M2a macrophages are profibrotic macrophages with opposite effects (Yue et al., 2020). The activation of M2-like macrophage-derived neuregulin-1/ERBB/PI3K/Akt signaling attenuates apoptosis and senescence of CFs in mice (Shiraishi et al., 2022). In infiltrated macrophages, a selective STING inhibitor H-151, alleviates cardiac fibrosis in the MI mouse model *via* the inhibition of cardiac dsDNA-triggered type I interferon response (Hu et al., 2022b). Furthermore, the knockdown of interferon-induced protein with tetratricopeptide repeats three in MI reduces the amount of CD68^+^ macrophages, TNF-α, IL-1β and IL-6 levels, infarct size, fibrosis, and collagen content (Sun et al., 2021a; Sun et al., 2021b). 5-methoxytryptophan reduces fibrosis by downregulating macrophages and T-cells infiltration (Hsu et al., 2021). Additionally, 2-benzylidene-3-cyclohexylamino-2,3-dihydro-1H-inden-1-one, the dual-specificity phosphatase six inhibitor, improves cardiac dysfunction and fibrosis in MI rats by inhibiting macrophages formation and inflammation after MI (Zhang et al., 2023). Taken together, the future studies might focus on modulating different populations and phenotypes of macrophages to improve patient prognosis and cardiac remodeling.

#### 4.2.2 Targeting the NLRP3 inflammasome

Several studies about NLRP3 inflammasome in the process of post-MI fibrosis have been reported. NLRP3 inflammasome is a proteolytic complex of the NLRP3 protein, procaspase-1, and apoptosis-associated speck-like protein (Zhang et al., 2022c), which regulates inflammatory response, pyroptosis and mitochondria and MF differentiation in cardiac fibrosis. Thus, it may represent a new therapeutical target, and its inhibitor oridonin decreases IL-1β and IL-18 levels and ameliorates myocardial fibrosis in MI mice (Gao et al., 2021). Moreover, calcium-sensing receptor activates the NLRP3 inflammasome in neutrophils and promotes apoptosis and fibrosis after MI, which is inhibited by Calhex231 (Liu et al., 2020b). Further, the N-butylidenephthalide-pretreated aging MI rats improves human adipose-derived stem cell engraftment and attenuates NLRP3 inflammasome-mediated cardiac fibrosis (Lee et al., 2020). Additionally, glycogen synthase kinase-3 inhibition suppresses the activation of NLRP3 inflammasome in CFs but not in CMs (Wang et al., 2020g). Therapeutic hypothermia attenuates MIRI *via* regulating sirtuin 3/NLRP3 signalling pathway (Zhang et al., 2022a). Thereby, the NLRP3 inflammasome is a key anti-fibrotic mediator, and its inhibition has beneficial effects on cardiac remodeling. Furthermore, its non-specific inhibitor colchicine is going on some clinical trials (Table 1).

### 4.3 Interventions for epigenetic regulation

#### 4.3.1 Histone acetylation and methylation

The challenge of epigenetic editing regarding specific cellular targets can provide promising therapeutic options in the coming years. Large preclinical studies have demonstrated the cardioprotective effects of histone deacetylase inhibitors *via* various mechanisms, such as the suppression of cardiac fibrosis, enhancement of angiogenesis and mitochondrial biogenesis, and prevention of electrical remodeling (Chun, 2020). Moreover, polyunsaturated fatty acids, eicosapentaenoic acid, and docosahexaenoic acid prevent cardiac remodeling by inhibiting p300-histone acetyl-transferase activity in MI rats (Sunagawa et al., 2022a), with the same efficacy as the inhibition of jumonji domain-containing protein three histone demethylase (Long et al., 2020). The inhibition of the disruptor of telomeric silencing 1-like expression reduces methylation modification of histone H3 on spleen tyrosine kinase promoter, which can inhibit the TGF-β1/smad3 axis and prevent myocardial fibrosis and CFs proliferation (Li et al., 2022a). Moreover, silence of methyltransferase-like 3 decreases m6A modification on fibrotic genes and reduces CFs proliferation and TGF-β1-induced collagen production (Li et al., 2021e).

#### 4.3.2 Intervention for ubiquitin

Conserved small molecular protein ubiquitin regulates protein turnover *via* the ubiquitin-proteasome system. Post-ischemic ubiquitin treatment attenuates cardiac dysfunction, myocardial fibrosis, apoptosis, hypertrophy, and serum cytokine/chemokine levels (Dalal et al., 2023), which suggests ubiquitin has a protective role in cardiac remodeling. Conversely, an endogenous E3 ubiquitin ligase ring-finger protein four knockdown induces extensive interstitial fibrosis after MI (Qiu et al., 2020). Additionally, ubiquitin C-terminal hydrolase L1 regulates cardiac fibrosis through glucose-regulated protein (Lei et al., 2020).

#### 4.3.3 Non-coding RNAs

The protein-coding genes are rare, meanwhile the majority of the transcribed genome are non-coding RNAs that mainly including microRNA, circular RNA (circRNA), long non-coding RNA (lncRNA). Numerous studies suggest that non-coding RNAs participate in pathophysiological process of post-MI fibrosis as epigenetic regulators, with the specialization of tissues and cells, so it is necessary to study the role of non-coding RNAs in fibrotic regulation and molecular mechanisms (Supplemental Table S1). In addition, non-coding RNAs can be carried into target cells by extracellular vesicles (EVs) (e.g., exosomes) with capacity to escape from immunogen clearance, and emerging studies show that they regulate fibrosis as diagnostic markers and therapeutic targets in MI (Table 2). With technical advances, extrusion filters produce massive EVs from live cells with native effects (Wang et al., 2021e) and the modulation of exosome imprinting repairs damaged tissue without immune rejection (Hill et al., 2022). Based on the above information, future research might be focusing on their biodistribution and precise delivery to target cells against cardiac fibrosis.

**TABLE 2:** The fibrotic modulatory effects of extracellular vesicles in myocardial infarction.

Extracellular vesicles	Model *in vitro*/vivo	Target gene/signaling pathway	The fibrotic modulatory effects	References
BMMSCs-derived-exosomal miR-29b-3p	MI rat model	ADAMTS16	cardiac fibrosis ↓	Zheng et al. (2022)
BMMSC-exosomes-miR-19a/19b	MI mouse model, hypoxic HL-1 cells	Bim and PTEN	cardiac fibrosis↓	Wang et al. (2020f)
BMMSCs-derived exosomes	MI rat model	EZH2/HMGA2/PI3K/Akt axis	cardiac fibrosis ↓	Jiao et al. (2022)
BMMSCs-derived EVs	AMI-induced HF rat model, hypoxic HUVECs	BMP2	cardiac fibrosis ↓	Xuan et al. (2022)
MSCs-EVs-miR-200b-3p	MI mouse model	BCL2L11/NLRP1	cardiac fibrosis↓	Wan et al. (2022a)
MSCs-derived EVs-miR-212-5p	clinical cardiac Samples, MI mouse model, CFs	NLRC5/VEGF/TGF-β1/smad	cardiac fibrosis ↓	Wu et al. (2022c)
telomerase/myocardin-coexpressing-MSCs	MI mouse model	miR-320a, miR-150-5p and miR-126-3p	myocardial revascularization ↑ tissue repair ↑cardiac fibrosis↓	Madonna et al. (2020)
EAT-secretory products	MI rat model, H9C2, CFs	EAT/miR-134-5p	cardiac fibrosis↑	Hao et al. (2021)
UMSC with β2 microglobulin deletion	MI rat model	Exosome/miR-24/Bim	cardiac fibrosis↓	Shao et al. (2020)
hAECs-derived exosomes	MI rat model	Exosomes	angiogenesis↑ apoptosis and fibrosis↓	Zhang et al. (2021c)
hPSCs--derived CPCs-EVs	MI mouse model; OGD-treated cardiomyocytes	LncRNA MALAT1/miR-497	cardiac fibrosis↓	Wu et al. (2020)
iPSC-derived CPC-EVs	MI mouse model	MiR-133-a1	expression of several pro-fibrotic genes↓ anti-fibrotic miR-133-a1↑	Lima Correa et al. (2021)
CBSC-derived exosomes	I/R mouse model, TGF-β1-treated CFs	small nucleolar RNA signaling	cardiac fibrosis↓	Schena et al. (2021)
Macrophages-EVs-circUbe3a	MI mouse model; CFs	miR-138-5p/RhoC	proliferation, migration, and phenotypic transformation of CFs↑ cardiac fibrosis↑	Wang et al. (2021f)
Macrophages-derived exosome-miR-21-5p	MI mouse model	Metalloproteinase 3	cardiac fibrosis↑	Dong et al. (2021a)
iCMs-exosomes-miR-181a	MI rat model; iPSC and iCMs	Sacubitril/Valsart inhibited exosomal miR-181a	cardiac fibrosis↓	Vaskova et al. (2020)
iCMs-exosomes	MI mouse model, hypoxic cardiomyocytes	the regulation of autophagy	cardiac fibrosis ↓	Santoso et al. (2020)

Note: ADSC, adipose-derived stem cells; ADAMTS16, a disintegrin and metalloproteinase with thrombospondin motifs 16; AT, adipose tissue; BCL2L11, Bcl-2–like protein 11; BMMSC, bone marrow mesenchymal stem cell; Bim, Bcl-2 interacting mediator of cell death; BMP2, bone morphogenetic protein 2; CBSC, cortical bone stem cell; CFs, cardiac fibroblasts; CPC, cardiovascular progenitor cells; CVPCs, cardiovascular progenitor cells; EAT, epicardial adipose tissue; EVs, extracellular vesicles; EZH2, enhancer of zeste 2 polycomb repressive complex 2 subunit; hAECs, human amniotic epithelial cells; HF, heart failure; HMGA2, high mobility group AT-hook 2; hPSCs, human pluripotent stem cells; HUVECs, human umbilical vein endothelial cells; iCMs, induced pluripotent stem cell-derived cardiomyocytes; I/R, ischemia/reperfusion; iPSC, induced pluripotent stem cell; MALAT1, metastasis-associated lung adenocarcinoma transcript 1; MI, myocardial infarction; MSC, mesenchymal stem cell; NLRP1, NLR family pyrin domain containing 1; NLRC5, nucleotidebinding and oligomerization domain-like receptor family Caspase recruitment domain–containing 5; OGD, oxygen-glucose deprivation; PI3K, phosphatidylinositol 3 kinase; PTEN, phosphatase and tensin homolog; TGF-β1, transforming growth factor β1; UMSC, umbilical mesenchymal stem cells; VEGF, vascular endothelial growth factor.

### 4.4 Regenerative medicine

Regenerative medicine is a challenging and broad interesting topic with enormous potential to regenerate novel cells by injecting cells, growth factors, and biomaterials and reprogramming, considering fibrotic scar structure and mechanics, to facilitate novel cell differentiation, maintenance, and function in the extracellular microenvironment (French and Holmes, 2019).

#### 4.4.1 Cell therapy

It’s a novel direction for anti-fibrotic treatment based on cell therapy in recent years. Given revascularization after MI as a cornerstone of current treatment, cell therapy seems like an ideal therapy. Because of the paracrine effects promoting repair of mesenchymal stem cells (MSCs), preconditioned MSCs *in situ* may be promising for post-MI repair (Sim et al., 2021). The paracrine therapeutic anti-inflammatory and antifibrotic effects of human amniotic MSCs are increased by S100A8/A9 and calcium-binding proteins after MI (Chen et al., 2021c). Moreover, subcutaneous implantation of TheraCyte devices encapsulating human W8B2+ cardiac stem cells improves cardiac remodeling and function after MI (Kompa et al., 2021), and stem cells-derived CMs patches restore normal electrical propagation without the risk of arrhythmia (Fassina et al., 2022). Induced cardiosphere (iCS) can be produced by self-replicative RNA approach, differentiating into CMs, while intravenous and intramyocardial injection of C-X-C chemokine receptor four positive subpopulation of iCS-derived cells has similar therapeutic effects in the mice MI model (Xu et al., 2021a). Additionally, intracoronary injection of allogeneic cardiosphere-derived cells immediately prior to reperfusion in an AMI pig model impedes adverse remodeling (Sousonis et al., 2021). The human amniotic membrane MSCs-derived conditioned medium modulates autophagy *via* mTOR/ULK1 pathway to against MIRI. (Mokhtari et al., 2021). The skeletal muscle-derived Sca-1+/PW1+/Pax7− interstitial cells attenuate cardiac remodeling after transplanted into the infarcted myocardium (Ruchaya et al., 2022).

#### 4.4.2 Cardiac reprogramming

Pluripotent stem cells generate functional CMs for post-MI regeneration, and cardiac reprogramming *in vivo* generates chamber-matched new CMs (Zhang et al., 2021d). Additionally, CFs can be reprogrammed into CMs and cardiovascular progenitor cells *via* lentiviral packaging techniques (Isomi et al., 2021) and CRISPR (Jiang et al., 2022), respectively. Moreover, microRNA-delivery platforms efficiently reprogram CFs into induced CMs (Yang et al., 2021a) or non-CMs into CMs-like cells (Kaur et al., 2021). Further, photo biomodulation therapy modulates gene transcription and miRNA expression to reverse the profibrotic signaling pathway (Feliciano et al., 2022), and fibroblast cilia regulates cardiac regeneration (Djenoune et al., 2022). A study has used miR-208b-3p mimic, ascorbic acid, and BMP4 to reprogram mouse tail-tip CFs into different cells (CMs, endothelial cells, and smooth muscle cells) and form cardiovascular tissue-like structure (Cho et al., 2021). Additionally, anti-BMP 1.3 monoclonal antibody inhibits the TGF-β pathway to reduce MFs activation and scar formation and exerts cardiac protection through BMP 5 (Vukicevic et al., 2022).

#### 4.4.3 Revascularization

As the main goal of treatment in MI, revascularization restores myocardial perfusion and reduces the infarction size, and improves cardiac function (Solhpour and Yusuf, 2014). PCI is the standard therapy for patients presenting in the first 12h from symptom onset, however, early thrombolysis should be considered with the absence of PCI (Leancă et al., 2022). Despite the above therapies, cardiac remodeling is still in one-third of MI patients (Solhpour and Yusuf, 2014). Macrophages and CFs also cause vascular disintegration and capillary rarefication (Wu et al., 2021b). Fortunately, the prevascularized cell sheets use reprogrammed cardiac cells to improve adverse remodeling (Song et al., 2020). High mobility group box one protein recruits bone marrow PDGFRα+-mesenchymal cells to induce angiogenesis and antifibrotic effect (Goto et al., 2020). However, these are basic studies without clinical applications. In a transmural scar distal to total coronary occlusion, up to 2 weeks after MI, large and medium coronary arteries maintain structural integrity, but are destroyed by subsequently progressive neointimal hyperplasia, intravascular fibrosis, and inward remodeling (Dedkov, 2021). Hence, the time frame for revascularization and optimizing cell-based regenerative therapies is the first 2 weeks in MI rats, but it is longer in humans (Dedkov, 2021).

#### 4.4.4 Biomaterials

Biomaterials (e.g., hydrogels, nanocarriers, and cardiac patches) support self-renewal in the injured heart, with reliable biosafety and moderate promise, on-demand biodegradation, and multiple biofunctions to deliver the therapeutic drugs; for instance, a type of hydrogel injected in the peri-infarcted zone promotes fibrotic healing in the infarct zone and inhibits reactive fibrosis and hypertrophy in the remote zone (Wen et al., 2020). Another injectable thermosensitive hydrogel of chitosan/dextran/β-glycerophosphate delivers umbilical cord MSCs to repair the damaged heart (Ke et al., 2020), while injectable disulfide-cross-linked chitosan hydrogel loaded with basic FGF synergistically exerts antifibrotic, antiapoptotic, and proangiogenic effects (Fu et al., 2022a). Moreover, a conductive and MMP-degradable hydrogel stabilizes hypoxia-inducible factor 1-α to reduce the infarcted area and inflammation factors, promote vascularization and the expression of junctional protein connexin 43, and recover cardiac function (Wei et al., 2022). The miR-124-3p-loaded nanoparticles activate PTEN/P13K/Akt pathway to decrease oxidative stress and myocardial injury (Cheng et al., 2022). However, there are still many matters that have not been figured out, such as the analysis of dyskinesia of the infarct zone, the mechanical properties of myocardium, and the key mechanisms underlying the cardiac benefits of a hydrogel implantation. Thus, hydrogels are still difficult to translate from preclinical studies to humans.

#### 4.4.5 Cardiac biomechanical modeling

Cardiac biomechanical modeling is a promising new tool for MI prognosis and therapy, such as a computational model predicting multiple time-dependent paracrine and intracellular drivers of CFs phenotype and post-MI fibrosis (Zeigler et al., 2020). Additionally, a library of scar tissue mechanical properties allows for the mechanics of cardiac modeling to assess the healing stage, rate, and collagen density, which can be potentially used as valuable biomarkers and therapies (Dempsey et al., 2020).

### 4.5 New strategies

#### 4.5.1 Traditional medication

Traditional medication has developed for thousands of years as an ancient wisdom, with various herbal drugs and their isolated compounds. Recently, some studies have been performed to evaluate the anti-fibrotic efficacy of traditional medication and compounds as innovative antifibrotic therapies. For example, both fasudil and aconite ameliorate myocardial fibrosis (Xiang et al., 2022; Xing et al., 2022). Danqi soft capsule inhibits CFs proliferation and migration, collagen secretion, and CF-to-MF transformation in post-MI HF rats (Ma et al., 2022). Many studies have demonstrated the signaling pathways of traditional medication. Calycosin and taohong siwu decoction suppress TGF-β1-induced CFs proliferation and collagen deposition (Tan et al., 2021; Chen et al., 2022a). Moreover, zerumbone, ganxin V, nutmeg-5, ginsenoside Rg2, jatrorrhizine, cardiotonic pill, and huoxin pill all regulate the TGF-β1/smad pathway to attenuate fibrosis (Li et al., 2020d; Wang et al., 2021b; Yan et al., 2022a; Yan et al., 2022b; Li et al., 2022c; Hao and Jiao, 2022; Liang et al., 2022). Moreover, citri reticulatae pericarpium also reduces CMs apoptosis, CFs proliferation, and CF-to-MF transformation by upregulating PPAR-γ expression (Chen et al., 2022c), while auraptene improves cardiac hypertrophy and dysfunction *via* activating PPARα (Sunagawa et al., 2022b). Tanshinone IIA suppresses inflammation and fibrosis *via* the regulation of NADPH oxidase 4 (Chen et al., 2021b). Additionally, panaxatriol saponin inhibits CFs activation and proliferation and fibrosis by regulating oxidative stress and Nrf2 pathway (Yao et al., 2022). Astragaloside IV improves fibrosis by suppressing ROS/Caspase-1/GSDMD pathway (Zhang et al., 2022d), and liquiritin play the same role *via* the inhibition of CCL5 expression and NF-κB pathway (Han et al., 2022). Storax effectively protects cardiomyocytes against myocardial fibrosis and cardiac dysfunction by inhibiting the AT1R/ankyrin repeat domain 1/p53 signaling pathway (Xu et al., 2022c). In addition, nutraceuticals (e.g., curcumin, berberine, hibiscus roselle, flaxseed, and garlic alliin) are useful as antifibrotic substances acting *via* multiple signaling pathways (Zivarpour et al., 2022).

#### 4.5.2 Exercise

In the modern era, unhealthy lifestyle (e.g., alcohol drinking, binge eating, smoking, physical inactivity) has been established as a risk factor for MI. Except for drug therapy, exercise-based cardiac rehabilitation should be taken into consideration to prevent the progression of adverse cardiac remodeling. For example, exercise significantly improves post-MI survival in the diet-induced obesity model (Peres Valgas Da Silva et al., 2022). Furthermore, moderate resistance exercise activates CMs proliferation through follistatin-like 1 (Hu et al., 2020). Moreover, exercise training increases FGF 21 and regulates the TGF-β1-smad2/3-MMP2/9 axis (Ma et al., 2021) and expression of lncRNAs H19, GAS5, and MIAT to decrease fibrosis in MI mice (Farsangi et al., 2021). Additionally, exercise inhibits tryptase release by mast cells and cardiac fibrosis (Bayat et al., 2021). The preconditioning with high-intensity interval training decreases heart injuries by increasing G-CSF and G-CSFR in the MI mouse model (Ghanimati et al., 2020). Noteworthily, the effect of combining the exercise with dietary intervention has been well validated. For example, aerobic-resistance training combined with vitamin D3 supplement suppresses the expression of TGF-β1, smad2/3, and collagen I and III to alleviate myocardial fibrosis and dysfunction (Mehdipoor et al., 2021).

#### 4.5.3 CAR-T cell therapy

Anti-fibrotic T-cell therapy with chimeric antigen receptor (CAR) is engineered receptors with function to redirect lymphocytes to recognize and eliminate cells expressing a specific target antigen. This interaction occurs in a specific CAR domain called “antigen binding domain” and allows endogenous activation of T cells, with subsequent elimination of target cells (Morfino et al., 2022). Aghajanian and his colleagues firstly investigated that T-cell immunotherapy could specifically target pathologic cardiac fibrosis. The endogenous cardiac fibroblasts target FAP has been shown to benefit cardiac fibrosis. Adoptive transfer of T cells expressing a CAR against FAP, results in a significant reduction in cardiac fibrosis and restoration of function after injury in mice (Aghajanian et al., 2019). Moreover, a new approach is represented by the use of CAR-T cells engineered *in vivo* using lipid nanoparticles containing mRNA coding for a receptor directed against the FAP protein, expressed by MFs (Rurik et al., 2022). This strategy has proved to be safe and effective in reducing myocardial fibrosis and improving cardiac function in mice. However, there are still many limitations to this approach. For example, it may lead to antigen escape and the syndrome of release of pro-inflammatory cytokines (Asnani, 2018; Majzner and Mackall, 2018; Ghosh et al., 2020). Thus, potential toxicity associated with CAR-T cell therapy has stimulated the search for alternative approaches, such as the use of CAR-natural killer cells, and safer cell programming methods. To sum up, the anti-fibrotic management following MI is still a serious challenge, and current therapies involving protecting the remaining CMs and preventing fibrosis.

#### 4.5.4 Intervention for metabolic abnormalities

Metabolism is an essential process for the maintenance of life, and metabolic homeostasis needs the coordination of anabolism and catabolism, which have been extensively studied. Recent studies involve metabolism of glucose, lipid, nucleotide, amino acid and so on. For example, glycolysis is not only related to TGF-β and Krüppel-like factor 5 signaling (Methatham et al., 2022), but also is inhibited by kallistatin/serpina3c to reduce post-MI fibrosis by activating Nr4a1 (Ji et al., 2022a). Protein kinase R regulates inflammation, insulin resistance, and glucose balance to improve cardiac fibrosis in isoproterenol-induced MI rats (Mangali et al., 2021). The high-density lipoproteins and stimulator of steroid receptor coactivators MCB-613 induce reverse remodeling *via* the reduction of cardiac dysfunction, hypertrophy, and fibrosis (De Geest and Mishra, 2021). Lysophosphatidic acid-lysophosphatidic acid receptor two signaling promotes angiogenesis and maintains vascular homeostasis to reduce scar formation and cardiac dysfunction (Pei et al., 2022). Furthermore, ketone ester reprograms gene expression of ketone body utilization and normalizes ATP production in post-infarct remodeling (Yurista et al., 2021). Sphingosine kinase one inhibitor PF543 reduces α-SMA, collagen, IL-1β, IL-6, and TNF-α levels to decrease myocardial injury, fibrosis, and inflammation (Wu et al., 2022a). Notably, deoxycholic acid-G protein-coupled bile acid receptor pathway activation also decreases inflammation and fibrosis (Wang et al., 2021a). Recombinant slit2, a secretive ECM protein, regulates the level of blood lipid decreasing total cholesterol, triglycerides, and low-density lipoprotein cholesterol, and increasing high-density lipoprotein cholesterol level in rats, which relieves the myocardial fibrosis, inflammation and oxidative stress in coronary heart disease (Liu et al., 2021a).

Following, we summarize the recent studies about amino acids and nucleotides. The inhibition of cardiac thyrotropin-releasing hormone reduces post-infarct hypertrophy and fibrosis (Schuman et al., 2021). Additionally, triiodothyronine pretreatment improves post-MI dysfunction and inhibits fibrosis by activating the insulin-like growth factor-1/PI3K/Akt signaling pathway (Zeng et al., 2021). In an MI sheep model, the upregulation of 5-hydroxytryptamine induces valve fibrosis, which could be improved by cyproheptadine (Marsit et al., 2022). Oral propionate, the important components of short-chain fatty acids, modulates macrophages polarization and pro-inflammatory cytokine *via* reducing JNK/p38/NF-κB phosphorylation to improve post-MI chronic cardiac remodeling (Zhou et al., 2023). The upregulated expression of ectonucleotide pyrophosphatase/phosphodiesterase 1 (ENPP1) after a cardiac injury can regulate cardiac repair, whereas uridine or ENPP1 inhibitor myricetin enhances cardiac repair by targeting the ENPP1/adenosine monophosphate (AMP) pathway (Li et al., 2022e).

Lastly, there are some other metabolic researches, for instance, chronic daily alcohol uptake enhances MI-induced cardiac dysfunction, fibrosis, and mitochondrial dysfunction (Liang et al., 2020). Urolithin A, a type of gut bacterial metabolite, inhibits myocardial fibrosis by activating the Nrf2 pathway (Chen et al., 2022d). Additionally, dimethyl fumarate promotes anti-inflammatory and preparative regulation by modulating oxidative metabolism in macrophages and CFs (Mouton et al., 2021). Furthermore, atypical chemokine receptor four deletion inhibits IL-6 expression and CFs proliferation to alleviate cardiac remodeling (Zhang et al., 2021a; Zhang et al., 2021b). Copper is reduced in myocardial ischemia-induced cardiac fibrosis, while it inhibits the CF-to-MF transformation as a pro-fibrinolytic switch and improves cardiac function (Xiao et al., 2023).

Post-MI cardiac fibrosis is related to unbalance of energy substrate metabolism (e.g., glucose, lipid, and amino acid). Furthermore, it is an energy-consuming process, which suggests that interventions of cardiac fibrosis combined with metabolic abnormalities are very important, with the positive efficacies against fibrosis.

#### 4.5.5 New pharmaceutical targets

An increasing number of new pharmaceutical targets have been developed, such as a soluble epoxide hydrolase vaccine, which improves cardiac function, and boron, as well as a new ligand of the apelin peptide jejunum receptor apela, which reduce myocardial fibrosis and apoptosis (Bouchareb et al., 2020; Pan et al., 2020; Kitsuka et al., 2022). There are also interventions targeting inflammation response, for instance, the inhibition of coagulation protein tissue factor cytoplasmic domain improves cardiac remodeling by regulating inflammation and angiogenesis (Chong et al., 2021). Resveratrol supplementation also decreases proinflammatory cytokine levels, cardiac dysfunction, and atrial interstitial fibrosis in MI-induced rats (Jiang et al., 2021).

Moreover, There are many other drugs that can improve fibrosis, such as piperine, thymoquinone, an oleanolic acid Qi-Tai-Suan, spinal cord stimulation and exogenous hydrogen sulfide (Li et al., 2020f; Viswanadha et al., 2020; Farag et al., 2021; Qian et al., 2022; He et al., 2023). Endostatin, alarin, L-carnitine, pentraxin 3 depletion, and mdivi-1 also can attenuate oxidative stress to inhibit myocardial fibrosis (Li et al., 2021b; Xu et al., 2021b; Emran et al., 2021; Xu et al., 2022a; Ding et al., 2022).

In experimental studies are useful to regulate cardiac fibrosis, even without clinical applications. For example, ephrinA1-Fc attenuates chronically non-reperfused post-MI remodeling (Whitehurst et al., 2020). Secreted frizzled protein three protects the heart *via* ischemic preconditioning in a pig model (Vatner et al., 2021). Upregulation of periostin regulates post-infarct fibrosis *via* cyclic AMP response element-binding protein 1 (Xue et al., 2020; Xue et al., 2022). Anti-proprotein convertase subtilisin/kexin type 9 intervention reduces infarct size and cardiac dysfunction (Guo et al., 2021). Epoxylipids improve cardiac fibrosis and dysfunction (Imig et al., 2022). Furthermore, menthol and HC-030031 reduces cardiac fibrosis *via* the regulation of cation channel (Li et al., 2020c; Wang et al., 2020e). As a pleiotropic hormone, serelaxin mitigates adverse remodeling and modulates bioactive sphingolipid signaling (Devarakonda et al., 2022). Additionally, nesfatin-1 suppresses necroptosis *via* regulating receptor-interacting protein kinase (RIPK) 1/RIPK3/mixed lineage kinase domain-like protein axis and RhoA/ Rho-associated coiled-coil-containing protein kinase/RIP3pathway (Sharifi et al., 2021). Pulsed-field ablation exerts ablating ventricular scar, and eliminates viable myocardium separated from the catheter by collagen and fat (Younis et al., 2022). Calpain inhibition decreases collagen formation (Potz et al., 2022). The purified human tropoelastin significantly repairs the infarcted heart in a MI rodent model (Hume et al., 2023).

Together with, researches on cardiac fibrosis have evolved with the advancement of various genetics and proteomics approaches in recent years. However, a lot of novel targets with anti-fibrotic effects are limited in basic researches, without clinical application. Advances have been made in the therapeutic field, and timely coronary reperfusion in associations with novel therapies, such as ARNI and SGLT2i, along with MRAs and beta blockers, counteract adverse ventricular remodeling and promote reverse ventricular remodeling, decreasing progression to HF and mortality. Future therapeutic perspectives, such as microRNAs, bone marrow derived-cells, and molecules targeting inflammation are currently under research, with promising results.

## 5 Conclusion

In summary, regional fibrotic control in infarcted areas and suppression of collagen accumulation in non-infarcted areas are vital to improve adverse remodeling and clinical outcome after MI. Even being treated according to the guideline-recommended protocols, it is still adverse fibrotic remodeling in MI patients. In the future, the study should focus on the exploring the deeper pathophysiological mechanisms underlying the onset and progression of post-MI fibrosis, then further determining therapeutic targets, and optimizing intervention strategies. In the meantime, antifibrotic precision interventions still need a clinical translation. Although clinical trials associated with anti-fibrotic drugs already have been performed, the patients included in trials are rather small. Hence, it is urgent to explore integrated and personalized therapeutic strategies to inhibit progressively fibrotic remodeling after MI. Moreover, early identification, diagnosis, and management of cardiac fibrosis are significantly important in improving the survival and prognosis of MI patients.
