# Supplementary material for: Post-myocardial infarction fibrosis: Pathophysiology, examination, and intervention
Source: Front Pharmacol. 2023 Mar 28;14:1070973. doi: 10.3389/fphar.2023.1070973 (PMC10086160; doi:10.3389/fphar.2023.1070973)
Supplement: Supplementary file 1 [file DataSheet1.pdf]

## Supplemental Table 1 The fibrotic modulatory effects of non-coding RNAs in myocardial infarction

| Non-coding RNAs | Model in vitro/vivo | Target gene/signaling pathway | The fibrotic modulatory effects | References |
|---|---|---|---|---|
| MicroRNA | | | | |
| miR-26a | STEMI patients, MI mouse model, OGD-treated H9C2 cells | ATM | cardiac apoptosis and fibrosis↓ | (Chiang et al., 2020) |
| miR-26b | MI rat model, CFs | HMGA2 | cardiac fibrosis↓<br>CFs activation ↓ | (Chen et al., 2020c) |
| miR-27a | MI mouse model, H/R-treated H9C2 cells | GFPT2/TGF-β/smad2/3 | cardiac fibrosis↓ | (Tian et al., 2021) |
| miR-29a-3p | MI mouse model, Ang II-stimulated CFs | TGF-β, collagen I and III in CFs | cardiac fibrosis ↓<br>CFs proliferation and migration↓<br>collagen synthesis ↓<br>MFs generation↓ | (Wang et al., 2021d) |
| miR-29b-3p | TGF-β1-treated CFs | FOS | cardiac fibrosis↓ | (Xue et al., 2020) |
| miR-30d | ischemic HF model, hypoxic CFs and ventricular myocytes | MAP4K4, integrin α5 | cardiac apoptosis and fibrosis↓ | (Li et al., 2021d) |
| miR-125b-5p | MFs-restricted AMPKα1 conditional knockout mouse with MI | connexin 43 | CFs proliferation and MFs content↑<br>connexin 43 in MFs↓ | (Dufeys et al., 2021) |
| miR-130a | MI mouse model, hypoxic CFs | TGF-β receptor 1/TGF-β /smad signaling pathway | cardiac fibrosis↓<br>CF-to-MF transformation↓ | (Feng et al., 2022) |
| miR-132 | rats with MI-induced HF,Ang II-treated CFs | PTEN /PI3K/Akt axis | cardiac fibrosis↓ | (Wang et al., 2020b) |
| miR-145 | MI model, hypoxic CFs | SOX9/Akt/GSK-3β/β-catenin | cardiac fibrosis ↓<br>CF proliferation, migration↓ | (Cui et al., 2021) |
| miR-155 | hypoxic CFs | SKIV2L2/TRA2A complex /circHIF1 α /fructose metabolism | miR-155 downregulation improves hypoxic injury | (Zhang et al., 2022f) |
| miR-210-3p | MI rat model | ADCY9 | cardiac fibrosis ↓ | (Wu et al., 2022d) |
| miR-218-5p | MI mouse model, TGF-β1-treated CFs | connexin 43 | cardiac fibrosis ↓ | (Sun et al., 2021a) |
| miR-221/222 | I/R mouse model, H/R-treated H9c2 cells, conditioned medium from ADSC | miR-221/222/p38/NF-κB mediated myocardial apoptosis via PUMA/p53/BCL2 axis and regulated fibrosis via ETS-1/fibronectin/collagen 3 axis | cardiac apoptosis and fibrosis↓ | (Lee et al., 2021) |
| circPAN3/miR-221 | MI rat model, TGF-β1-treated CFs | circPAN3/miR-221/FoxO3/ ATG7 | cardiac fibrosis↑ | (Li et al., 2020a) |
| miR-195 | MI rat model | TGF-β1/smad | cardiac fibrosis↑ | (Wang et al., 2020a) |
| LncRNA | | | | |
| lncRNA MHRT | MI mouse model, TGF-β1-treated CFs | miR-3185 | cardiac fibrosis↑ | (Lang et al., 2021) |
| lncRNA NORAD | MI rat model, hypoxic H9C2 | miR-577/COBLL1 | cardiac fibrosis↑ | (Xiong et al., 2021) |
| lncRNA TUG1 | MI rat model, AngII-treated MFs | TUG1/miR-133b/CTGF | proliferation and collagen generation of MFs↑ | (Zhang et al., 2021b) |
| lncRNA SNHG7 | MI mouse model, CFs | miR-34-5p/ROCK1, miR-455-3p/PAFR | cardiac fibrosis↑ | (Wang et al., 2020c) |

| | | | | |
|---|---|---|---|---|
| lncRNA SAIL | MI mouse model, TGF-β1-treated CFs | SAFB | cardiac fibrosis↓ | (Luo et al., 2021) |
| lncRNA 00152 | MI patients and mouse model, CFs | smad7 | CFs proliferation, migration↑ | (Song et al., 2021b) |
| lncRNA Ang362 | MI rat model, TGF-β1-treated CFs | smad7 | cardiac fibrosis↑ | (Chen et al., 2020a) |
| lncRNA_000898 | MI mouse model, hypoxic cardiomyocytes | miR-375/PDK1 | cardiac fibrosis↓ | (Yan et al., 2020) |

Note: ADCY9, adenylyl cyclase type 9; ADSC, adipose-derived stem cells; AMPK, adenosine monophosphate-activated protein kinase; ATG7, Autophagy Related 7; ATM, ataxia–telangiectasia mutated; α-SMA, α-smooth muscle actin; BCL2L11, Bcl-2–like protein 11; BMP2, bone morphogenetic protein 2; CPCs, cardiovascular progenitor cells; CBSC, Cortical bone stem cell; CFs, cardiac fibroblasts; FoxO3, forkhead box O-3; GFPT2, glutamine fructose-6-phosphate transaminase 2; GSK-3β, glycogen synthase kinase 3 β; HF, heart failure; H/R, hypoxia/reoxygenation; HUVECs, human umbilical vein endothelial cells; HMGA2, high mobility group AT-hook 2; I/R, ischemia/reperfusion; MFs, myofibroblasts; MAP4K4, mitogen-associate protein kinase 4; MHRT, myosin heavy chain associated RNA transcript; MI, myocardial infarction; STEMI, ST-segment elevation myocardial infarction; NORAD, non-coding RNA activated by DNA damage; OGD, oxygen-glucose deprivation; PAFR, platelet-activating factor receptor; PTEN, phosphatase and tensin homolog; PI3K, phosphatidylinositol 3 kinase; PUMA, p53 upregulated modulator of apoptosis; ROCK, Rho-associated, coiled-coil domain containing protein kinases; SNHG7, small nuclear RNA host gene 7; SAFB, scafold attachment factor B; SAIL, scafold attachment factor B interacting lncRNA; SOX9, sex-determining region Y box 9; TGF-β1, transforming growth factor β1; TRA2A, transformer 2 alpha; TUG1, taurine upregulation gene 1.
